# Direction-selective retinal ganglion cells encode motion direction uniformly, despite having discretely distributed cardinal preferences

**DOI:** 10.1371/journal.pcbi.1014872

**Published:** 2026-11-02

**Authors:** Carlo Paris, Félix Hubert, Felix Franke, Olivier Marre, Matthew Chalk, Ulisse Ferrari

**Affiliations:** 1 Institut de la Vision, Sorbonne Université, Paris, France; 2 Department of Basic Neurosciences, University of Geneva, Geneva, Switzerland; 3 Institute of Molecular and Clinical Ophthalmology Basel, Basel, Switzerland; Johns Hopkins University, UNITED STATES OF AMERICA

## Abstract

On-Off direction-selective retinal ganglion cells (DS RGCs) exhibit broad tuning curves, responding robustly to motion aligned with or near their preferred direction. These cells comprise four major subtypes, each tuned to motion along one of the four cardinal axes: nasal, superior, temporal, or inferior. However, natural stimuli can move in any direction, and it remains unclear whether intermediate directions are encoded less effectively, or whether this cardinal organization nevertheless supports uniform direction encoding. Here, we combined previous electrophysiological recordings of DS cells presented with a moving light bar stimulus with an information-theoretic measure, the Stimulus Specific Information, to estimate the directional sensitivity of small populations of recorded neurons. This analysis revealed that DS RGC populations are uniformly sensitive across all directions of motion. We then asked whether the observed homogeneous sensitivity was a consequence of DS RGCs maximizing the average stimulus information. Simulations with artificially modified tuning curve widths revealed that DS cells prioritize avoiding pronounced drops in sensitivity over maximizing the average transmitted information. This property reduces large deficits in sensitivity for certain directions, thereby limiting potential “blind spots” in the encoding of motion. More generally, our results raise the possibility that maintaining a high minimum sensitivity could be one factor influencing the organization of sensory representations.

## Author summary

The detection of moving objects by the visual system starts in the retina. A special set of retinal ganglion cells, called direction-selective cells, respond most strongly when objects move along one of four main directions (up, down, left, or right). But in real life, motion can occur in any direction. What does the sensitivity of this system look like for individual directions of motion? For example, does the retina miss some information for diagonally moving stimuli?

By studying small groups of these cells, we found that together they provide nearly uniform sensitivity to all possible directions of motion. In this work we show that this balance in sensitivity is achieved by optimizing the width of the cells’ tuning curves. Interestingly, the cells are not tuned to maximize the total amount of information they could carry. Instead, they are tuned to make sure that no direction of motion is too poorly represented.

Our results suggest that the retina prioritizes reliable coverage of all directions over maximum efficiency. This strategy ensures robust motion detection in everyday vision, even if it sacrifices some potential information capacity.

## Introduction

Direction selectivity is one of the fundamental tasks of visual perception, evidenced by the presence of direction-selective (DS) cells in the visual system of a wide variety of species ranging from zebrafish larvae [1] to macaques [2]. DS cells respond robustly to motion aligned with or near their preferred direction, are among the most extensively studied types of cells, and have been observed in cat, ferret and macaque cortex [3–5], as well as salamander, mouse, rabbit and, recently, macaque, retina [2,6–8], to name a few. A striking difference between retinal and cortical DS cells is that, whereas the cortical cells exhibit a continuous spectrum of all preferred directions, DS retinal ganglion cells (RGCs) exhibit preferred directions that are restricted to a limited set of discrete axes. Given this difference, it remains unclear how functional organization of preferred directions impacts coding.

On-Off DS RGC respond strongly to both the onset and offset of a contrast change in their receptive fields if an object is moving along their preferred direction of motion [8,9]. Furthermore, rabbit On-Off DS RGCs feature 4 preferred directions, roughly corresponding to cartesian axes: nasal, ventral, temporal and dorsal [10]. Intermediate directions of motion cause intermediate spiking with a slow monotonic decrease as the direction of motion moves away from the preferred one, resulting, in the rabbit retina, in broad tuning curves [11,12]. Previous studies found a correspondence between these directions and the directions of apparent motion across the retina produced by contractions of the 4 rectus muscles, and suggested that these cells might control optokinetic nystagmus [10,13,14]. However, real-world visual stimuli can move in any direction, and from a coding perspective, it remains unclear whether motion along intermediate directions is poorly encoded by retinal DS cells.

Noting the small number of roughly equally spaced preferred directions, we inquire what the sensitivity to different stimuli looks like for a direction-selective system characterized by only four preferred directions. For example, do the large firing rates associated to stimuli aligned with the preferred directions lead to better encoding of those movements? In that case the system’s sensitivity would suffer a decrease for directions between the 4 preferred ones. Or, conversely, are intermediate directions better encoded than the preferred ones due to multiple On-Off DS RGCs of different types firing at intermediate capacity?

In order to estimate the system’s sensitivity to any direction of motion, we use information theory to link stimulus and neural response. Information theory [15] has a long history of application to neural systems and many studies successfully used measures such as the Mutual Information to estimate the encoding capacity of sensory neurons [16–18] (for review see [19]). However, Mutual Information is an average quantity and does not reflect sensitivity to individual directions of motion. To address this issue, here we use the Stimulus Specific Information (SSI) [20,21], a decomposition of the Mutual Information which quantifies the contribution of individual stimuli.

A key result of this paper is that the tuning curve widths of On-Off DS RGCs maximize the minimum sensitivity, rather than maximizing the average sensitivity (i.e., the Mutual Information). To demonstrate this result, we applied an information-theoretic analysis to previous retinal recordings in rabbit [22,23]. We begin by introducing and characterizing the dataset of electrophysiological recordings of DS RGC responses to repeated presentations of a moving bar stimulus. For quadruplets of DS RGCs - each comprising one cell per type - we observe that sensitivity is approximately uniform across motion directions. Then, by artificially varying the widths of the tuning curves, we find that DS RGCs prioritize maximizing the minimum sensitivity across all possible directions of motion. In other words, the widths and preferred directions of the population are arranged such that the system avoids having any motion directions that are encoded significantly worse than others, thereby preventing the emergence of a kind of “blind spots”. We show that the cells exhibit tuning curve widths associated with near-optimal coding performance, rather than the theoretical optimum predicted by our framework, suggesting that factors beyond the optimization criterion considered here may contribute to shaping their tuning properties. Our results stem from the findings of [22] by recasting the problem within an information-theoretic framework, linking the observed tuning widths to a MaxMin coding principle that generalizes Efficient coding and explains the near-uniform sensitivity across motion directions. Finally, our crawling procedure explains the discrepancy between empirical and theoretically optimal tuning widths, revealing a near-optimal regime.

## Materials and methods

### Experimental data

The experimental protocols has been previously presented, see [22,23] for more details. Briefly, the data was recorded from New Zealand albino rabbit retinas in the course of 7 experiments. Each retina was extracted and placed ganglion cell side down on a high-density multi-electrode array featuring 11011 electrodes [24]. The light stimulation was performed using a light bar moving in one of 36 possible equidistant directions, each repeated 100 times. Cell activity was monitored online in order to find electrodes recording On-Off Direction Selective cell activities. Spike sorting was done manually using the UltraMegaSort2000 [25]. The spike sorted cells were subsequently classified as On-Off DSRGCs according to the Direction Selectivity Index (DSI) and the On-Off Index (OOI), respectively defined as:


$$\begin{array}{c} DSI=\frac{\left|\left|\sum_{i=1}^{N}{\vec{v}}_{i}\right|\right|}{\sum_{i=1}^{N}\left|\left|{\vec{v}}_{i}\right|\right|} \end{array}$$

$$\begin{array}{c} OOI=\frac{LE-TE}{LE+TE} \end{array}$$

where ${\vec{v}}_{i}$ is the cell’s average firing rate in the $i_{th}$ direction, while *LE* and *TE* denote the responses in the cell’s preferred direction of motion to the leading and trailing edge when the bar is moving in the cell’s preferred direction of motion. Only those cells were kept which had *DSI* > 0.2 and |OOI|<0.8. A total of *n* = 90 cells satisfied these requirements.

### Fitting the tuning curves

We model the tuning curves with a Flat-Topped Von Mises (FTVM) function, an extension of the regular Von Mises function (rVM) [23,26,27]. Its functional form reads:


$$\begin{array}{c} f(\theta {)}_{TC}=\frac{A}{Z(s,g)}\exp^{\frac{\cos(\theta -\mu -g\cdot \sin(\theta -\mu ))}{s}}+B \end{array}$$ (1)

where *Z*(*s*,*g*) is a normalization constant. The independent variables of the function are the directions of motion θ∈Θ. The remaining quantities are fitting parameters: μ is the cell’s preferred direction, *B* is the cell’s activity in the anti-preferred direction, *A* is the area above the baseline, *s* is the so-called concentration parameter which regulates the curve’s width (akin to the standard deviation in the 1-dimensional Gaussian case) and *g* is the flatness parameter. By setting *g* = 0, we can retrieve the regular Von Mises function. Reducin*g g* results in a function which is more peaked than the *g* = 0 case, whereas increasing it flattens the top.

An additional selection of cells is done at this point by keeping only those cells for which the Mean Square Error of the FTVM fit is less than 15, and those whose FTVM fit features a Full Width at Half Maximum less than 180°. Out of the initial 90 cells, 6 do not satisfy the *MSE* < 15 criterion and an additional 2 do not satisfy the *FWHM* < 180° criterion, and are therefore excluded from subsequent analyses.

### Noise model

The conditional probability p(r|θ), and therefore the $I_{SSI}$ itself, depends on the choice of noise model. We assume a Poissonian noise model so that both the spiking average and variance for any stimulus θ are given by the corresponding value of the tuning curve $f_{TC}(\theta )$, that is we assume the rate $\lambda =f_{TC}(\theta )$. With these assumptions, the conditional probability reads


$$\begin{array}{c} p(r|\theta )=\frac{f_{TC}(\theta {)}^{r}}{r!}\cdot \exp(-f_{TC}(\theta )) \end{array}$$ (2)

This is broadly supported by the data and at any rate seems to be an acceptable approximation (Fig ~ F in S1 Text).

Finally, unless stated otherwise, we assume conditional independence of the responses, meaning that for a quadruplet of cells responding to a stimulus θ∈Θ, the probability of responses $\left(r_{1},r_{2},r_{3},r_{4}\right)$ is equal to


$$\begin{array}{c} p(r_{1},r_{2},r_{3},r_{4}|\theta )=\prod_{i=1}^{4}p(r_{i}|\theta )=\prod_{i=1}^{4}p(r_{i}|f_{TC}^{i}(\theta )) \end{array}$$ (3)

### A measure of sensitivity - stimulus specific information

This paper studies how the width of On-Off Direction Selective Cell tuning curves impacts the sensitivity of this system to various directions of motion. We approach this problem from an information-theoretic perspective and use the Stimulus Specific Information (SSI) as a measure of sensitivity [20,21], a decomposition of the Mutual Information over the stimulus ensemble:


$$\begin{array}{c} I_{SSI}(\theta )=\sum_{r\in R}p(r|\theta )\cdot \left\{H\left[\Theta \right]-H\left[\Theta |r\right]\right\} \end{array}$$ (4)

where θ∈Θ is the light bar’s direction of motion, $R\in {\mathbb{N}}_{0}$ is the cell response ensemble and *r* the spike count vector of the cells. p(θ) is the distribution of stimuli (here uniform), while p(r|θ) is the probability that a given response *r* is elicited by a particular stimulus θ and H[·] are entropies. The term H[Θ]−H[Θ|r] is the information gained about which stimulus was shown to the system upon learning that the system responded with *r*. The SSI is the average information gained by the system’s response, weighed by the conditional probability that a response was elicited by a particular stimulus. It bears an explicit dependence on the stimulus θ∈Θ and its average over the stimulus ensemble equals the Mutual Information between the two ensembles, MI[Θ;R]:


$$\begin{array}{c} E[I_{SSI}{]}_{p(\theta )}=\sum_{\theta \in \Theta }p(\theta )\cdot I_{SSI(\theta )}=MI[\Theta ;R] \end{array}$$ (5)

### Quadruplet formation

We consider as elementary computational units quadruplets of cells such that each cell belongs to one of the four subtypes according to their preferred direction of motion (superior, temporal, inferior, nasal). Such a choice follows from the fact that the receptive fields belonging to each of the 4 subtypes completely tile the retina independently from cells of the other 3 subtypes, with minimal overlap of same-subtype receptive fields [28,29]. Consequently a small stimulus falling on a point of the entire visual field will most likely be directly processed by 4 On-Off direction selective retinal ganglion cells, namely 1 belonging to each of the 4 subtypes. Within every experiment, all possible combinations of 4 cells are taken while preserving the original sub-typing. Cells from different experiments are not mixed. The result is a total of *N* = 648 quadruplets.

### Tuning curve stretching simulation

Each cell’s FTVM fit has 5 parameters (Eq), one of which is the concentration parameter *s* which controls the tuning curve’s width. A quadruplet of cells will correspondingly have 4 such concentration parameters and an average Full Width at Half Maximum.

In our simulation, in order to obtain a “new” quadruplet we multiply the 4 *s* parameters of the original empirical quadruplet by the same multiplier. All other (16) parameters are kept constant. Multiplying the original *s* parameters by a value lower or higher than 1 results in respectively narrower or wider tuning curves. Therefore by choosing an array of multipliers spanning values from 0.01 to 10 we are able to explore a wide variety of avatars of the original quadruplet, each differing from the others by their average Full Width at Half Maximum.

For each such quadruplet we calculate the associated Sensitivity as a function of the direction of motion, its minimum sensitivity $\min_{\theta \in \Theta }SSI(\theta )$ and average sensitivity E[SSI(θ)] (=MI[Θ;R]). Since the computational complexity of an exact calculation of the SSI increases exponentially with the number of cells, we implemented a Monte Carlo sampling method as described in [30]. Doing this for all multipliers (and thus all width regimes) yields the behavior of the minimum and average sensitivities as a function of the quadruplet’s average width.

## Results

### On-Off direction selective retinal ganglion cells

We aim to quantify the sensitivity to motion in all possible directions of On-Off DS RGCs. We use previously recorded data collected from ex-vivo New Zealand White rabbit retinas [22,23]. Spiking activity was recorded from *N* = 82 On-Off DS RGC, responding to bars moving in 36 equidistant directions, across 7 experiments (Methods). The cells show wide, bell-shaped tuning curves and can be typed according to their preferred directions, which are distributed around the 4 cardinal axes of the body - nasal, superior, temporal, and inferior (Fig 1A and 1B).

**Fig 1:**
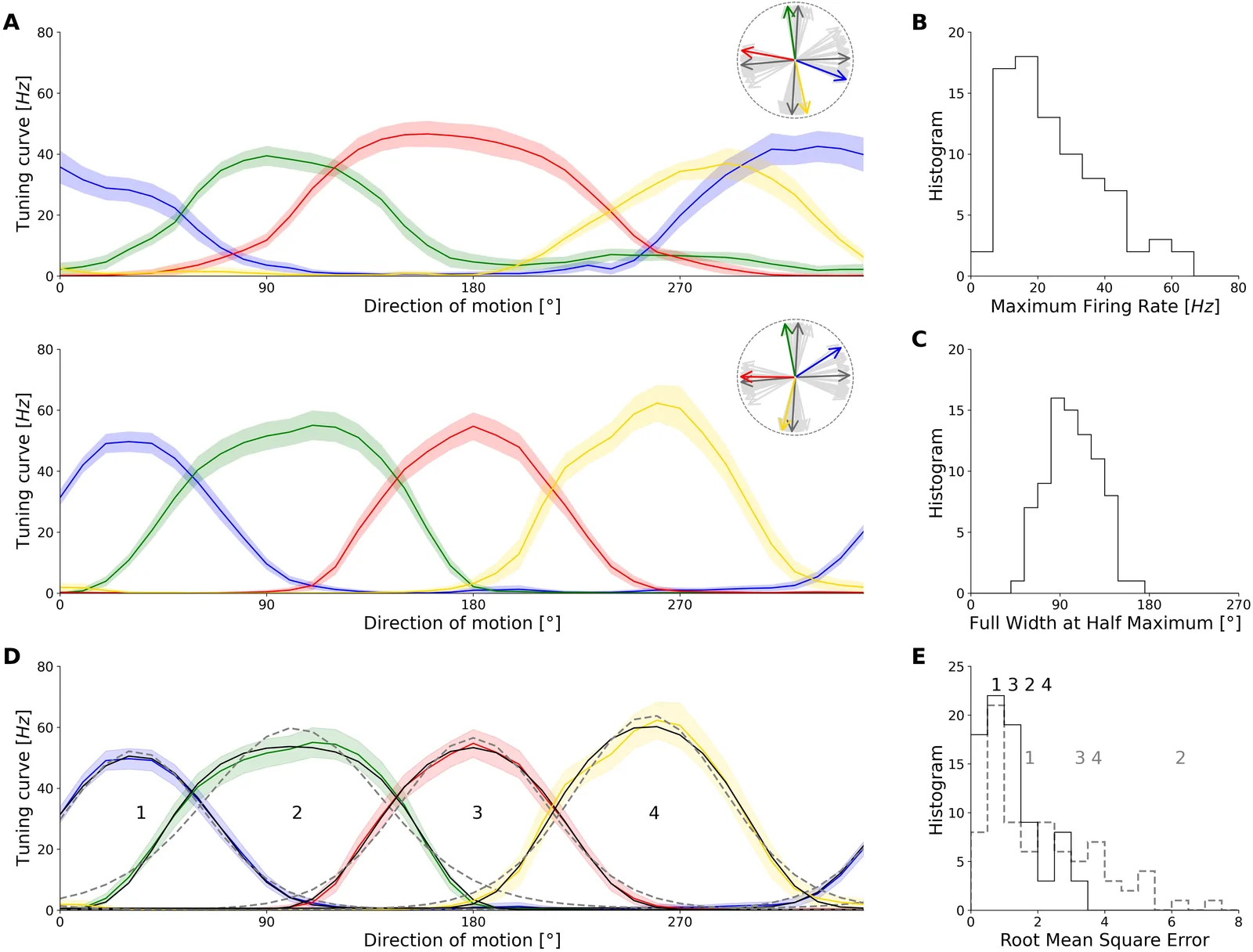
Analysis and parametrization of On-Off DS RGC tuning curves. **(A)** Two example quadruplets of On-Off Direction Selective Retinal Ganglion Cells’ tuning curves. Each cell belongs to one of four types identified through their preferred directions (nasal - blue; superior - green; temporal - red; inferior - yellow). Solid lines and colored strips show the firing rates’ average and standard deviation, respectively, over 100 trials for each direction of motion. Inset: distribution of all the cell’s preferred directions (light gray) and the average preferred direction for each type (dark gray) compared to the preferred directions belonging to the cells in plot (colored). **(B)** Distribution of maximum firing rates over *N* = 82 cells. mean±std=40±22 **(C)** Distribution of tuning curves’ widths, calculated as Full Widths at Half Maximum. $mean\pm std=103^{\circ }\pm 26^{\circ }$. **(D)** Comparison between regular Von Mises (dashed black line, rVM) and Flat-Topped Von Mises (solid black line, FTVM). The commonly used rVM fit fails to reproduce the tuning curves’ curvatures near the maximum and the baseline firing rates. On the other hand, the FTVM manages to do so remarkably well, best exemplified by tuning curve 2. **(E)** Comparison of Root Mean Square Error distributions between data and fitted tuning curves for the rVM (dashed) and FTVM (solid) fitting functions. The numbers 1-4 reference the tuning curves from panel D, and show the RMSE reduction in switching from a rVM to a FTVM fit.

Cells exhibit a wide distribution of maximal firing rates, ranging from 10*Hz* to 80*Hz*, with most cells peaking at ~20*Hz* (40±22Hz, Fig 1C). Notwithstanding the diversity of maximal firing rates, the cells’ tuning curves, when normalized, resemble one another, as evidenced by the fact that most of the cells have full widths at half maxima (FWHM) close to 100° ($103^{\circ }\pm 26^{\circ }$, Fig 1D). Additionally, the tuning curves overlap noticeably for orthogonal preferred directions (Fig 1A, 1B and 1D), and because of this we expect the sensitivity of a population of DS RGCs to behave very differently from the sensitivity associated with individual cells.

### The sensitivity of small populations of On-Off DS RGCs is homogeneous across directions

Real-world stimuli move in all directions, yet DS RGCs exhibit preferred directions which align along only four axes. This raises the question of whether populations of these cells maintain uniform sensitivity across all directions of motion, or whether the encoding of certain directions is significantly compromised due to this specific distribution of preferred directions. To test this, we consider combinations of 4 cells - one cell for each type - as units of computation. This approach is motivated by the fact that On-Off DS RGCs tile the retina such that receptive fields of cells with the same preferred direction exhibit minimal overlap, whereas receptive fields of different types overlap randomly [28]. Consequently, at any given point on the retina, a small stimulus is processed by only four cells, one from each type.

We form quadruplets by exhaustively combining tuning curves of different types within individual experiments. For each quadruplet we calculate its stimulus sensitivity using the *Stimulus specific information* (Fig 2A and 2B, Methods and [20,21]. We choose this quantity because it measures the reduction of stimulus uncertainty (entropy) after observing the cells’ response and for this data it aligns with decoding performance better than other quantities (Supplementary Section Fig ~ A in S1 Text). Additionally, its average over the stimulus ensemble yields the Mutual Information between stimulus and response [20], and this allows us to to compare individual stimulus sensitivites to overall performance.

**Fig 2:**
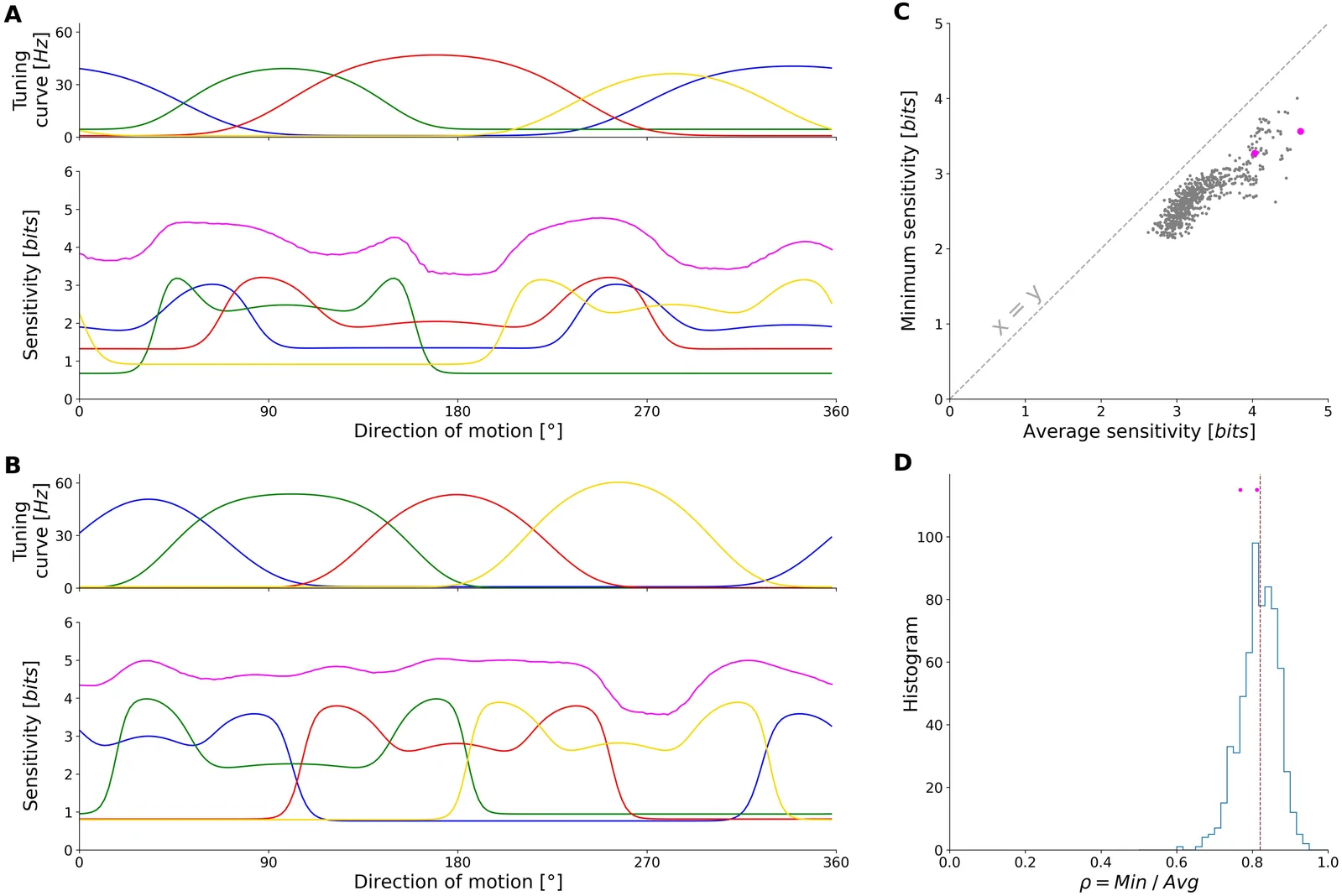
The sensitivity of quadruplets of On-Off DS RGCs is homogeneous across directions. **(A)** Upper - fits for tuning curves from Fig 1A; lower - associated individual sensitivity (non-magenta colors) and population sensitivity (magenta). **(B)** Analogous to A, but for the quadruplet in Fig 1B. **(C)** Average sensitivity versus Minimum sensitivity for each randomly formed quadruplet (N = 648). Magenta points indicate the sensitivities of quadruplets from A and **B.** Equality line identifies cases where the sensitivity is constant, with *Min*(*SSI*) = *Avg*(*SSI*). **(D)** Distribution of quadruplets’ minimum sensitivities relative to the respective averages (dotted line shows the median). 439 out of 648 quadruplets (67.7%) show a minimum sensitivity greater than 80% of the average.

As previously observed, the stimulus sensitivity of individual cells is large around their preferred directions (Fig 2A and 2B), with typical peaks corresponding to the tuning curves’ flanks [21]. On the other hand, quadruplet sensitivity expresses the stimulus information encoded by a group of four cells, and in the examples presented it appears fairly homogeneous (magenta lines in Fig 2A and 2B). Upon obtaining the sensitivities for all quadruplets, we notice that the minimum of the sensitivity often results close to the average value (Fig 2C) - the mutual information. This indicates that the sensitivities of the quadruplets oscillate conservatively around the average. More than half of the quadruplets show a minimum sensitivity value within 20% of the average (Fig 2D). The difference between any given quadruplet’s Mutual Information and minimum sensitivity lies between 0.5 and 1 bit of information.

A flat sensitivity is achieved through a balance between maximal spiking of a cell for stimuli moving near its preferred direction, on the one hand, and multiple cells spiking at intermediate levels when the stimulus lies between preferred directions, on the other hand. Populations of four DS RGCs thus exhibit uniform sensitivity to all possible directions of motion. This property should result from the specific shape and width of the tuning curves. Therefore, in the next section we address how overall stimulus sensitivity - and its directional uniformity - depends on the shape of individual tuning curves.

### Widths of On-Off DS RGC tuning curves are tuned to maximize sensitivity minima

The minimum of the sensitivity is associated with the least informative, or most poorly encoded, stimulus. Increasing this value would be advantageous for the system as it would simultaneously eliminate a potentially fatal “blind spot” and heighten the overall average. On the other hand, the average sensitivity corresponds to the Mutual Information *MI* [20], a measure of the average amount of information the system can transmit. Previous works argued that sensory systems evolved to maximize this quantity under energetic constraints, a hypothesis known as the Efficient coding principle [31–33]. However, as maximizing the average or overall sensitivity might leave the aforementioned “blind spots.” Given the small differences between quadruplets’ Mutual Information and minimum sensitivities (Fig 2D) we hypothesized that the cells have tuning curve widths which yield sensitivities as constant as possible along the stimulus ensemble. To test this hypothesis, we simulate how each quadruplet’s sensitivity changes upon modifying the constituent tuning curve widths (Methods) and focus on minimum sensitivity and Mutual Information.

For each modified width of a given quadruplet, we calculate the quadruplet’s minimum sensitivity and the corresponding Mutual Information (average sensitivity) (Fig 3A). We can plot the last 2 quantities against the associated average tuning curve widths whereupon we obtain curves which, as expected, drop towards 0 at the extremes. Indeed, as the tuning curves become wider and wider, the cells’ activities become increasingly uniform across directions of motion, ultimately losing direction selectivity altogether (Fig 3A, right). On the other hand, as the tuning curves become narrower they tend towards peaked functions centered in the system’s 4 preferred directions of motion. In those directions, the encoding is perfect (so the sensitivity equals the stimulus entropy), but other directions are completely overlooked (leading to null sensitivity). In that case the minimum of the sensitivity goes to zero (Fig 3A left).

**Fig 3:**
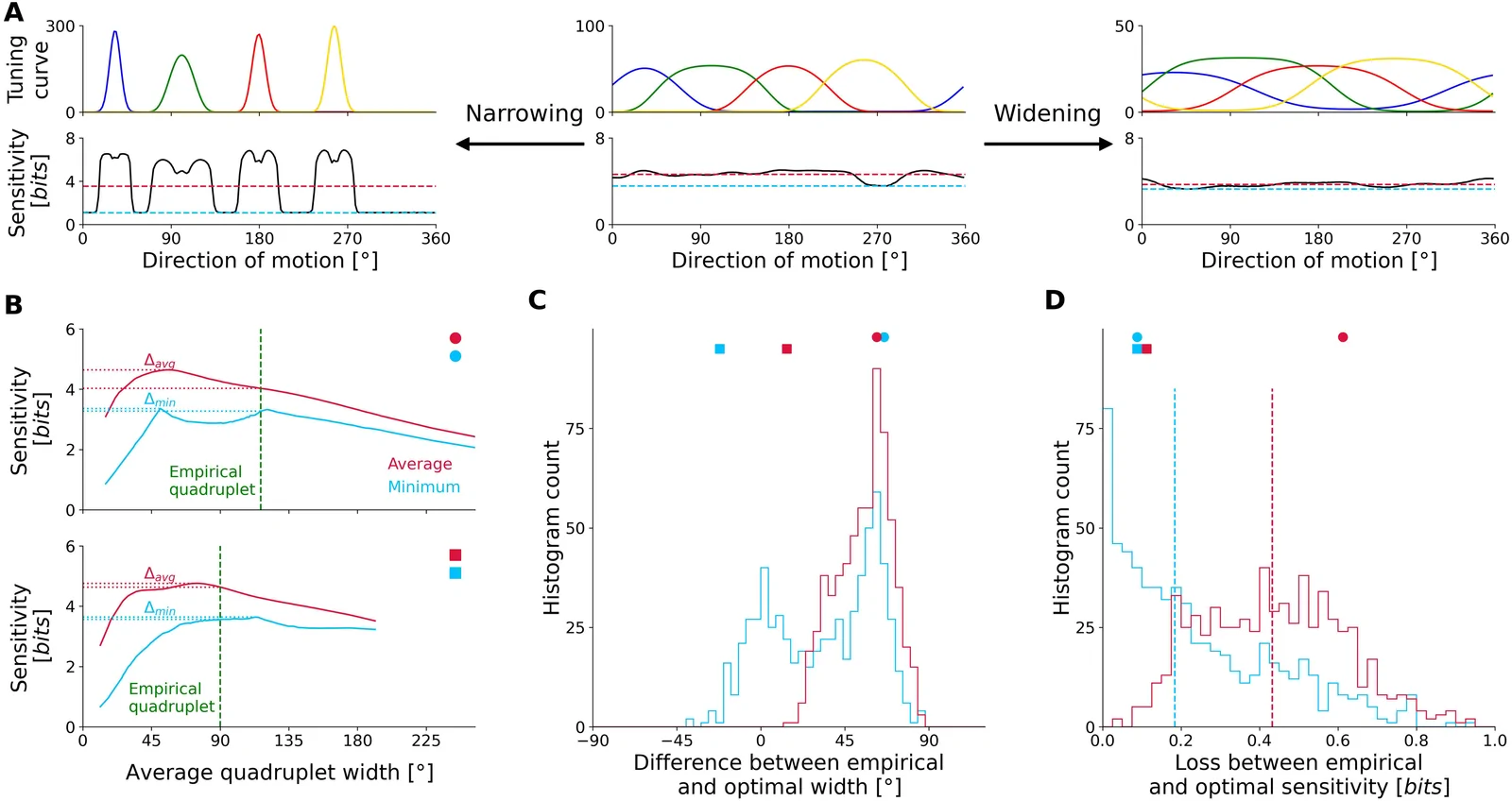
Empirical tuning curve widths achieve a near-optimal minimum sensitivity. **(A)** Simulation of various tuning curve width regimes. Starting from empirically obtained widths, quadruplets of “synthetic” avatars are generated by modifying the cells’ widths to smaller or larger values. **(B)** Average (red) and minimum (blue) sensitivity as a function of the stretched average tuning curve width for the quadruplets from Fig 1A and 1B. The green line is the average Full Width at Half Maximum (FWHM) of the 4 empirical tuning curves forming this quadruplet. $\Delta_{avg}$ is the difference between the maximum average sensitivity and the average sensitivity achieved by the empirical quadruplet (red). $\Delta_{min}$ is analogous, but for the minimum sensitivity (blue). **(C)** Histograms of differences between the average width of a given empirical quadruplet and the average width at which this quadruplet maximizes either the average (red) or the minimum sensitivity (blue). A positive difference means the empirical quadruplet is wider than its maximizing avatar. The circle and square symbols mark the bins where the quadruplets from panel B fall. **(D)** Histograms of $\Delta_{avg}$ (red) and $\Delta_{min}$ (blue). These quantities express a loss with respect to the case in which the quadruplet maximizes either the average or the minimum. The dashed lines indicate the histograms’ median values. Note how the $\Delta_{min}$ median is smaller than that of $\Delta_{avg}$, indicating that empirical quadruplets have an almost optimal minimum sensitivity.

By performing simulations of systematic compression and broadening of the tuning curve widths, we observe two different behaviors for minimum sensitivity and Mutual Information. The minimum sensitivity can present either 1 or 2 clearly visible local maxima (exemplified in Fig 3B, blue lines). These maxima occur at tuning-curve widths that are either substantially narrower than the empirical values (left peak in the upper panel of Fig 3B) or close to the empirical widths (right peak in the upper panel of Fig 3B). Consistently, when studying all the *N* = 648 possible quadruplets, the histogram of the widths corresponding to the optimal minimum sensitivity is bimodal, with peaks occurring at widths similar to the empirical ones (left peak of blue histogram in Fig 3C) and widths occurring at much narrower widths (right peak of blue histogram in Fig 3C). Although the globally optimal average tuning curve width for a given quadruplet may be much narrower than the empirical one, the resulting difference in minimum sensitivity is typically small. In fact, more than half of the empirical quadruplets exhibit a loss of less than 0.2 *bits* relative to the globally optimal (i.e., highest) minimum sensitivity (blue histogram in Fig 3D; median = 0.184 *bits*). Overall, these results indicate that the widths of the empirical tuning curves are tuned to values that yield near-optimal minimum sensitivity for the quadruplets of cells.

In contrast, we observe a different outcome for the Mutual Information (average sensitivity). For most quadruplets, we observe a single maximum (Fig 3B, red lines), typically occurring at tuning curve widths much narrower than the empirical ones (Fig 3C, red histogram). Accordingly, the difference between empirical and optimal average sensitivity is often substantial, with only about 10%of quadruplets showing a loss smaller than 0.2 bits (Fig 3D, red histogram, median = 0.433 *bits*).

These results suggest that empirical quadruplets are tuned to maximize minimum sensitivity rather than Mutual Information: a substantial number of quadruplets have an empirical width that achieves a performance close to the local maximum along the minimum sensitivity, while at the same time leading to a large loss in terms of average sensitivity (Mutual Information). Nevertheless, many quadruplets seem to reach a near-optimal minimum sensitivity, rather than achieving the globally optimal configuration. Next, we wonder whether this solution represents a compromise by incurring a marginal loss in performance in order to avoid an optimization toward very narrow widths.

### Widths of On-Off DS RGC tuning curves are “good enough”

For about half of the quadruplets, the width that maximizes the minimum sensitivity is narrower than the empirical one (Fig 3C), and is comparable to the width that maximizes average sensitivity. Yet the empirical quadruplet exhibits much broader tuning curves while still incurring only minor losses of performance in terms of minimum sensitivity. Motivated by these findings, we test whether these broader, but “good enough”, widths could result from an optimization that starts from unspecific cells and gradually sharpens their tuning.

For each quadruplet, we start from an avatar system with very large widths, and progressively narrow them by crawling leftwards along the curves of either the minimum sensitivity or the Mutual Information (Fig 4A), stopping at the first local maximum of the curve (Methods). The example quadruplet in the top panel of Fig 3A illustrates a case where the “crawling” procedure converges to an optimal minimum sensitivity that, while globally sub-optimal, closely matches the empirical one (Fig 4A top). In contrast, for the quadruplet shown in the bottom panel of Fig 3A, the same procedure yields the globally optimal width, identical to the one found earlier (Fig 4A bottom). In both cases, optimization of the Mutual Information converges to a single, global maximum (Figs 3A and 4A).

**Fig 4:**
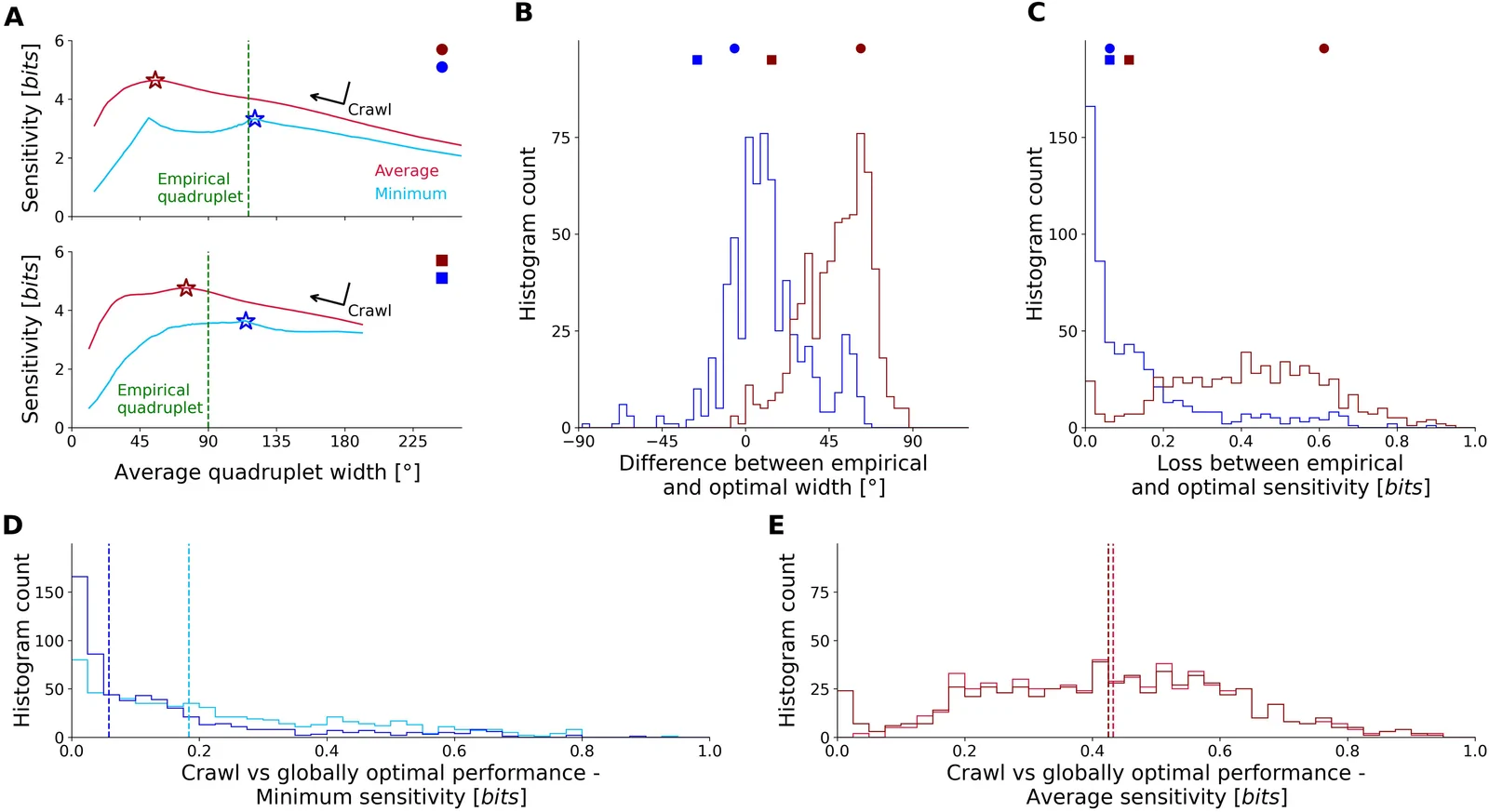
The crawl procedure along curves of minimum sensitivity as function of average quadruplet tuning curve widths identifies tuning curve widths close to the empirical widths. **(A)** For each quadruplet, starting from large average tuning curve widths (far right) and crawling leftward, we find the first local maximum along either the minimum or average sensitivity curve. The darkred and darkblue stars mark the 2 resulting peaks. **(B)** Similar to Fig 3C, histogram of the difference between their average width in the empirical regime and the quadruplet’s width yielded by the crawling procedure. The latter may or may not correspond to the globally optimal width. Circles and squares mark the quadruplets from panel **A. (C)** Histogram of performance losses between the sensitivities in the empirical regime and the optimal sensitivities reached by the crawling procedure. **(D)** Comparison between the performance losses estimated by finding the global maximum of the minimum sensitivity (Fig 3D, blue) versus the losses of minimum sensitivity estimated through the crawl procedure (panel D, darkblue). The dashed lines mark the distributions’ medians: dark blue 0.06 *bits*, light blue 0.18 *bits*. **(E)** Analogous to panel D, but for the average sensitivity.

An analysis including all possible quadruplet combinations confirms these results. Our crawling procedure for the minimum sensitivity produces widths that closely match the empirical ones (Fig 4B and 4C, blue histograms). Consistently, the performance losses with respect to the empirical width of the crawl optimum is smaller than those of the global one (Fig 4D). Conversely, applying the crawling procedure to the Mutual Information converges to the same optimal width identified previously (compare red histograms in Figs 3C and 4B), with correspondingly large performance losses. Regardless of possible underlying mechanisms or interpretations (see Discussion), our results indicate that empirical widths lie near a local optimum of minimum sensitivity - one that can be reached by progressively sharpening initially broad, directionally non-selective tuning curves.

### Sensitivity is uniform and MaxMin principle is observed under altered stimulus conditions

In the dataset analyzed so far, the stimulus consisted of a moving light bar with leading-edge widths of 0.5 *mm* and 1 *mm*, moving at 1.6mm/s in 36 equally spaced directions. To test the robustness of our results, we analyzed an additional dataset of HDMEA electrophysiological recordings from New Zealand albino rabbit On-Off DS RGCs, acquired across three additional, independent experiments. In this new dataset, the moving light bar had a leading-edge width of 0.5 *mm*, moved at 0.8mm/s, and was presented in 16 equally spaced directions. Each direction was repeated 100 times per experiment. Across the three experiments, 30 cells were recorded, of which 23 satisfied our inclusion criterion of a Flat-Topped Von Mises fit Mean Squared Error below 15. Because two of the three experiments lacked at least one preferred-direction class, complete quadruplets could not be formed within individual experiments. We therefore generated 270 quadruplets from the pooled population of 23 cells.

Fig 5A shows the tuning curves and associated directional sensitivity profile of an example quadruplet from the new dataset. Fig 5B shows the distribution of uniformity indices (minimum divided by average sensitivity) for the 270 quadruplets. Consistent with the previously analyzed dataset, the uniformity index remained high, with a median value of approximately 0.76, and the distributions from the two datasets substantially overlapped.

**Fig 5:**
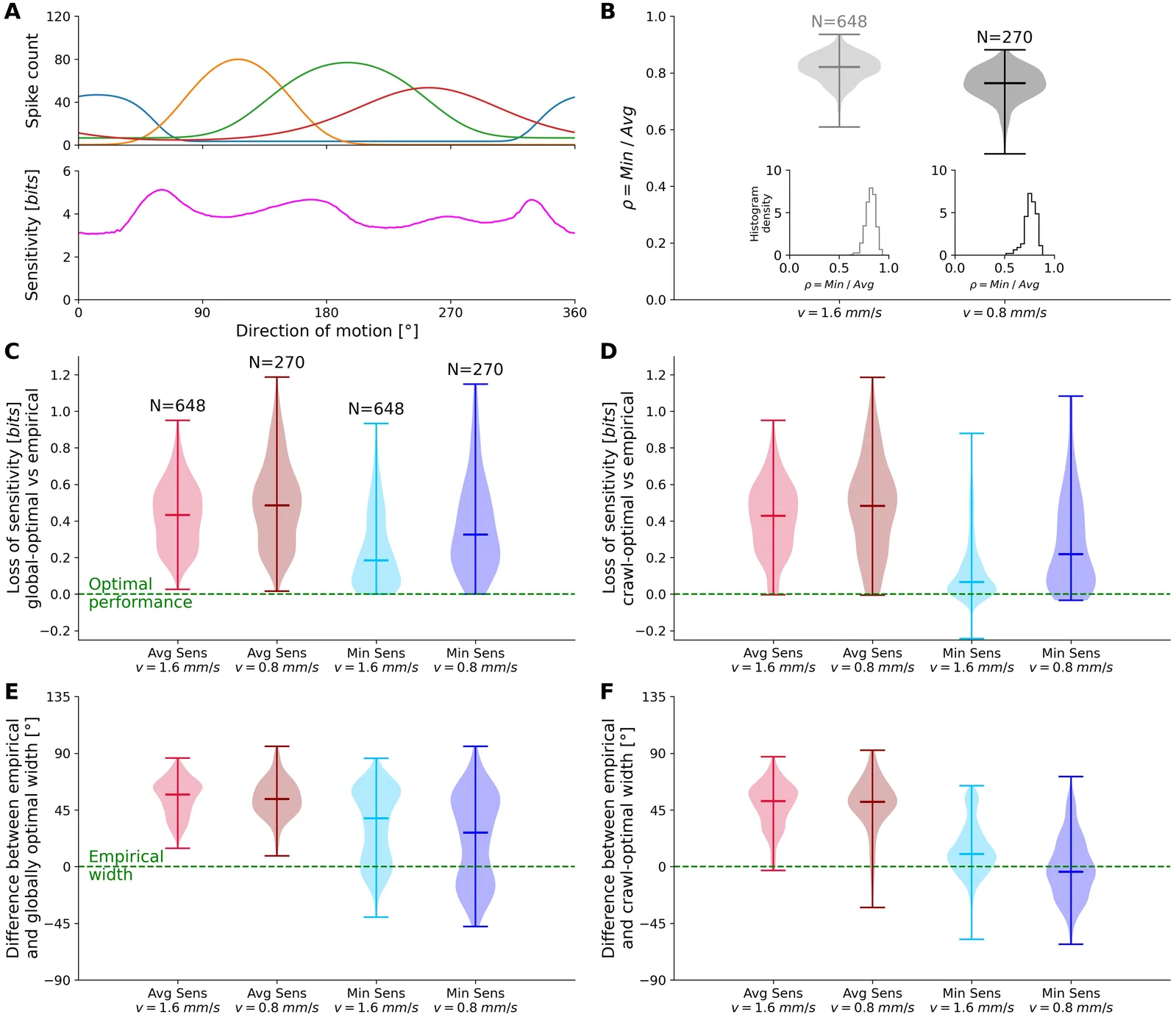
Uniform sensitivity and the MaxMin principles can still be observed in quadruplets of On-Off DS RGC cells exposed to slower moving stimuli. **(A)** Example quadruplet from the new HDMEA dataset recorded using a light bar moving at 0.8mm/s in 16 directions, together with its associated sensitivity profile. **(B)** Uniformity index distributions from the previously analyzed and present dataset substantially overlap. **(C)** Violin plots showing sensitivity losses of empirical quadruplets relative to globally optimal solutions for minimum (blue) and average (red) sensitivity. Light colors correspond to the previous dataset (bar velocity v=1.6mm/s), dark colors to the present dataset (bar velocity v=0.8mm/s). In both datasets, empirical quadruplets are closer to the optimum for minimum sensitivity than for average sensitivity. **(D)** Sensitivity losses relative to crawl-optimal solutions, showing substantially smaller deviations from empirical performances than globally optimal solutions. **(E)** Differences between empirical tuning widths and widths achieving globally optimal sensitivity. Average sensitivity is optimized at narrow widths, whereas minimum sensitivity also exhibits optima near empirical widths. **(F)** Differences between empirical widths and crawl-optimal widths, showing convergence toward empirical widths when optimizing minimum sensitivity.

Fig 5C compares the performances of empirical quadruplets with globally optimal solutions in terms of minimum and average sensitivity. Losses relative to the global optimum were consistently smaller when evaluated using minimum sensitivity than average sensitivity. In the dataset with velocity v=0.8mm/s, the minimum sensitivity loss distribution was more strongly skewed toward zero than the average sensitivity distribution, indicating that empirical quadruplets more closely maximize minimum sensitivity than average sensitivity. Median losses in the new dataset were approximately 0.35 *bits* for minimum sensitivity and approximately 0.5 bits for average sensitivity. Fig 5D repeats the comparison using crawl-optimal rather than globally optimal solutions. As in the main text, crawl-optimal performances were substantially closer to the empirical observations than globally optimal performances, particularly when evaluated using minimum sensitivity.

Panels E and F of Fig 5 compare empirical tuning widths with the widths maximizing sensitivity under globally optimal and crawl-optimal solutions, respectively. For both the previous and present dataset, globally optimal average sensitivity was associated with narrow tuning curve widths, whereas minimum sensitivity exhibited a bimodal structure, with optima occurring either at very narrow widths or near empirically observed widths. Importantly, the crawl algorithm combined with the MaxMin principle converged toward empirical tuning widths, with the crawl-optimal minimum-sensitivity distributions concentrated near zero difference from the biological widths.

Together, these results demonstrate that sensitivity uniformity, the MaxMin principle as a coding strategy for On-Off DS RGC populations, and crawl-based recovery of empirically observed tuning widths remain robust across different stimulus statistics.

## Discussion

In summary, we investigated direction selective retinal ganglion cells (DS RGCs) from rabbit retinas to determine whether the discrete distribution of four equidistant preferred directions results in homogeneous stimulus sensitivity - and whether this organization may be justified by an optimization principle. To address this question, we quantified the sensitivity using a decomposition of the Mutual Information along the stimulus ensemble and found that randomly assorted quadruplets of On-Off DS RGCs exhibited uniform sensitivity across directions of motion. We then showed, through simulations across widths, that the empirical tuning curve widths place the cells in a regime that doesn’t optimize Mutual Information (average sensitivity), but instead yields minimum sensitivities close to the globally optimal ones. Despite this, the empirical widths were rarely globally optimal with respect to minimum sensitivity, suggesting that the cells operate in a “good enough” regime that results from an optimization that gradually sharpens the tuning curves (Fig 4).

We observed that empirical widths lie close to a local maximum of the minimum sensitivity, rather than the global maximum, which occurs at sharper tuning curves. From a developmental standpoint, although widths narrower than the empirical ones may yield higher minimum sensitivities (Fig 3C), achieving them may be unfavorable due to biological constraints. Biological widths are arrived at through a gradual development of asymmetric synaptic connections between Starburst amacrine cells and DS RGCs during the organism’s postnatal maturation [34,35]. In rabbits and mice, the distribution of preferred directions and the tuning curve widths have been observed to develop after eye opening, with preferred directions aligning progressively with the canonical axes and tuning curves becoming narrower [36]. If synaptic asymmetry underlies direction selectivity, then an initially symmetric connectivity would be associated with completely flat, directionally non-selective tuning curves. As development proceeds, the emergence of asymmetry would lead to reduced firing in the anti-preferred direction, accompanied by a gradual relative increase in response near the final preferred direction. Optimizing the tuning curve width beyond a certain level of performance likely incurs for the system some form of energy and wiring strength cost which may outweigh the benefits.

Although our study focuses on the role of tuning curve widths, other parameters may also contribute to achieving optimality. Coding performance depends on the spacing of preferred directions, with equal spacing enabling optimal information encoding, and deviations from this arrangement leading to reduced performance. Theunissen et al. [37] showed that sliding one tuning curve while keeping others fixed leads to a monotonic decrease in performance, reaching a minimum when the sliding tuning curve overlaps with one of its neighbors. Our results are compatible with these findings, as the vacancy created by the shifted tuning curve would significantly reduce sensitivity, thereby violating the Max-Min principle. Similarly, the placement and number of preferred directions also impacts coding, as shown in [22] using the same dataset as the present study. While perfectly spaced and more numerous preferred directions lead to lower decoding error, Fiscella et al. propose that the upgrade might not be conducive to a net improvement, considering the resource cost necessary to achieve it. From a functional perspective, our results complement those of [22]: their analyses also point to uniform decoding performance and favor tuning widths that minimize the worst-case error, consistent with our Max-Min principle. By adopting an information-theoretic encoding framework, we further connect these findings to efficient coding theory and show that they are robust across stimuli and prior distributions. Our results regarding uniform sensitivity for a system with cells selective to a discrete number of preferred stimuli align with Theunissen et al. [37], even though they worked on a completely different biological system (the cricket cercal system). Notably, both systems strive to maximize the minimum sensitivity, supporting this goal as a general encoding principle to understand the organization of sensory systems.

Technically, Theunissen et al. [37] measured sensitivity with a different decomposition of the Mutual Information. However, both their chosen measure and ours can be related to the stimulus dependent decoding error $\sigma_{dec}(\theta )$ through the same functional form (see Appendix of [37] and Fig ~ A in S1 Text, and Protocol 2 of Butts and Goldman [21] for a more detailed description of how different information-based sensitivity measures compare to each other). We chose to work with a different measure - the Stimulus Specific Information [20,21] - because it quantifies the residual uncertainty when decoding the stimulus from the neuronal responses, making it particularly well-suited for investigating how individual stimuli are represented. In our opinion, there is no single right decomposition of the Mutual Information, and the choice should depend either on interpretability or the specific system function one wishes to study, following criteria such as additivity [38], causality [20], coordinate invariance [39] and locality [40].

Several simplifying assumptions were made in this study that may give rise to differences between the biological system of On-Off DS RGCs and the effective system considered in our analyses. First, cell quadruplets were assembled without accounting for the spatial arrangement of receptive fields. Because the tuning properties of a given cell may be influenced by neighboring cells, it is important to assess whether position-dependent interactions affect the results reported here. An analysis of uniformity of sensitivity and optimality splitting quadruplets made by proximal cells from the others did not revealed any significant difference between the two groups (Fig ~ B in S1 Text). Second, retinal neurons in-vivo are exposed to a broad range of stimulus statistics, which in our framework are reflected in the prior distribution over stimuli. Throughout this work, we assumed a uniform prior, corresponding to equal probabilities for all directions of motion. An important extension would be to investigate the robustness of our findings under non-uniform stimulus priors [41]. For quadruplets of On-Off DS RGCs, our results indicate that the likelihood distribution (i.e., the tuning curves) provides the dominant contribution to sensitivity, while the prior distribution affects sensitivity only through a constant additive shift determined by the prior entropy (Fig ~ C in S1 Text). Finally, a stimulus sweeping over the retina will most likely activate many DS cells at once. Therefore, a complete understanding of On-Off DS RGCs would require extending the present analysis beyond quadruplets of cells and investigating the sensitivity of larger neuronal populations. Similar behavior was observed in simulations of populations of 8 and 16 cells, which showed largely the same sensitivity trends as quadruplets, suggesting that our main conclusions may generalize to larger populations (Fig ~ D in S1 Text). Finally, previous work on the same dataset [22,23] has shown that noise correlations can impact stimulus encoding. However, our analysis assumes conditional independence between neurons within each quadruplet. Although noise correlations may impact coding, their effect (Fig ~ G in S1 Text) was rather constant for different directions. As a result, noise correlations minimally affected both the uniformity of sensitivity as well as the relationship between minimum and average sensitivity.

In the future it will be interesting to see how well the Max-Min sensitivity principle holds up in other systems. For instance, On RGCs in the rabbit retina have been shown to be characterized by 3 roughly equidistant preferred directions [10,14,41]. We could wonder if also their tuning curve width is explainable through the same principle. To explore this hypothesis, we applied our stretching and crawling analysis (Figs 3 and 4) to a system with 3 DS cells (Fig ~ E in S1 Text), with parameters estimated from the measurement reported in [42]. Our results are consistent with the trends observed in quadruplets and point in the same direction as our proposed framework. A refinement of this analysis is consigned to future work. Additionally, equivalent questions might be posed for cortical cells, such as DS cells in the primary visual cortex which share the same one-dimensional stimulus ensemble with their retinal counterparts, or even place cells whose stimulus ensemble is at least two-dimensional. Confronting these questions will pave the way to a more complete understanding of the role specific cell types play in perception, and the Max-Min principle can serve as a compass to guide that research.

## Supporting information

S1 TextSupplementary analyses of DS RGC sensitivity.This section presents analyses of effects of measurement quantity, receptive field proximity, assumed prior, population size and cell type. **Fig A The Stimulus Specific Information offers a good measure for the stimulus sensitivity.** **(A)** upper - Example quadruplet of tuning curves from Fig 1A; middle - Population sensitivity of Fig 1A quadruplet in magenta and its Fisher information FI(θ) in orange; bottom - Decoding errors estimated from the sensitivity, Fisher Information or a Bayesian unbiased decoder. The sensitivity error is proportional to $e^{-SSI(\theta )}$ with the *SSI* expressed in *nats*. The Fisher error is obtained from the unbiased Cramér-Rao bound as 1$\sqrt{FI(\theta )}$, the inverse square root of the Fisher information. We assume a flat prior p(θ)=1|Θ|. Notice the Fisher information peaking around 180°, in correspondence to the red cell from the uppermost panel. This is due to the slopes of all 4 tuning curves being vanishingly small in that area, resulting in a Fisher Information error that tends to extremely large values where the Bayesian decoder error is bounded. Because the Fisher information is small, the decoding bias is large [43,44] and the unbiased Cramér-Rao bound is violated. **(B)** Same as in A, but for Fig 1B. **(C,D)** Some quadruplets display a Fisher information that, due to their particular configuration of tuning curve slopes, tend to zero in certain points. Consequently the associated decoding error estimates grow to values which egregiously exceed the Bayesian decoding error. **(E)** Spearman Rank correlation coefficients between the Bayesian decoding error and either $e^{-SSI(\theta )}$ (magenta) or 1$\sqrt{FI(\theta )}$ (orange). We can see that for *N* = 648 quadruplets the Sensitivity error estimate correlates with the Bayesian error more than the Fisher information error does. **(F)** Difference between Sensitivity and Fisher performance in terms of Spearman Rank coefficients of correlation to the Bayesian decoding error. The differences are done quadruplet-by-quadruplet, showing that for all of them the Sensitivity performance correlates with the Bayesian error more than the Fisher performance does. **Fig B Impact of receptive field proximity on sensitivity of On-Off DS RGCs.** **(A)** Uniformity distributions for quadruplet subpopulations with non-proximal (np-RFs, black) and proximal receptive fields (p-RFs, green). **(B)** Violin plots of minimum sensitivity losses for empirical quadruplets relative to global (light) and crawl (dark) optima. Proximal quadruplets show larger losses at empirical average tuning widths compared to non-proximal quadruplets, whereas crawl optima converge near empirical performance regardless of receptive field position. **(C)** Violin plots of width differences for global and crawl optima, separated by receptive field overlap. Global optima for proximal quadruplets favor narrow tuning widths, while non-proximal quadruplets display a more bimodal distribution; crawl optima remain close to empirical widths in both cases. **Fig C Effect of prior probability.** **(A)** Simple geometric model of how the probability distribution of directions of motion changes with eccentricity. The arc represents a quadrant of the retina, the blue arrows represent the distribution of directions of motion in the retina’s geometric center (equiprobability is represented by identical arrow length), while the pink arrows represent the modified non-uniform prior at an eccentricity of $\approx 75^{\circ }$. **(B)** The uniform and non-uniform priors obtained through the model in panel A, shown in blue and pink, respectively. **(C,D)** The quadruplets with the lowest and highest average spike counts, respectively, from our population of quadruplets. **(E,F)** The sensitivities of the weakest and strongest quadruplets, computed using the uniform prior (blue) and the non-uniform prior (pink). The adjusted sensitivity (black) is obtained by shifting the pink curve by the difference between the uniform and non-uniform prior entropies, to highlight the overlap with the uniform prior sensitivity and the contribution of the likelihood in its computation. **(G,H)** The minimum sensitivities as a function of average tuning curve width (obtained as in Fig 3 of the main text), computed using the uniform and non-uniform priors. The black lines are obtained using the non-uniform prior and then adjusted as in panels E and F. **Fig D Sensitivity of populations composed of 8 and 16 On-Off DS RGC.** **(A)** Example 8-cell population: two neurons per preferred direction (upper) and corresponding sensitivity profile (lower). **(B)** As in panel A, but for a 16-cell population with four neurons per preferred direction. **(C)** Uniformity indices for 4-, 8-, and 16-cell populations (blue, orange, green). Variability in uniformity decreases with increasing population size. **(D)** Minimum (blue) and average (red) sensitivity losses relative to global optima. Empirical populations are closer to optimal under the minimum-sensitivity objective. **(E)** The crawl algorithm converges toward empirical configurations along the minimum-sensitivity landscape, while identifying non-empirical global optima under the average-sensitivity objective. Although average-sensitivity optima deviate further from empirical configurations, the crawl still recovers empirical minimum-sensitivity solutions. **(F)** Distributions of differences between empirical tuning widths and globally optimal widths for 8- and 16-cell populations mirror those of quadruplets. Narrow widths optimize average sensitivity, whereas minimum sensitivity is achieved across a broad range of widths, many close to empirical values. **(G)** The crawl algorithm recovers empirically consistent tuning widths along the minimum-sensitivity landscape for 4-, 8-, and 16-cell populations. **Fig E Sensitivity simulation for triplets of ON cells yields similar, albeit less marked, results as for quadruplets.** **(A)** Upper: example of a triplet of tuning curves; middle: example of individual and population sensitivities associated to example triplet; Bottom: behavior of triplet sensitivity average and minimum (respectively lightred and lightblue) as function of simulated tuning curve widening. **(B)** Difference between the estimated average “biological” triplet width and the width associated to the global maximum of the average and minimum sensitivity (lightred and lightblue respectively), analogous to Fig 3C. There appears to be little difference between the average triplet widths that maximize the minimum sensitivity and the average sensitivity. Most of the maxima occur at tuning curve widths approximately 45° narrower than the estimated biological width. The colored markers identify the bins containing the example triplet from panel A. **(C)** Histograms of performance loss of estimated biological minimum and average sensitivity compared to the associated maxima (respectively lightblue and lightred), analogous to Fig 3D. The trend is similar to that of Fig 3D, but with a markedly smaller peak around zero of the minimum sensitivity histogram. **(D)** Difference between the estimated average “biological” triplet width and the width associated to the crawl-obtained maximum of the average and minimum sensitivity (darkred and darkblue respectively), analogous to Fig 4B. While the histogram associated to the average is largely the same as the on in Fig 4B, the histogram associated to the minimum, although centered in zero, is much wider than its quadruplet counterpart. **(E)** Histograms of performance loss of estimated biological minimum and average sensitivity compared to the associated crawl-obtained maxima, analogous to Fig 4C. As for quadruplets, the crawl-obtained widths favor maximizing the sensitivity minimum rather than the average, although the result is less marked in the case of cell triplets. **Fig F Relationship between spike count average and variance for On–Off DS RGCs.** Each point represents the mean and variance of a cell’s spike count when presented with some direction of motion. The plot contains data from *N* = 90 cells and |Θ|=36 directions. The dashed line indicates Poisson variability (*E*[*x*] = Var[*x*]). The data follow the Poisson prediction for low to moderate spike counts, but show increasing dispersion for larger mean spike counts. Nevertheless, the dispersion is approximately symmetric with respect to the identity line, suggesting that assuming an over- or under-dispersed variability model would not substantially alter our conclusions. **Fig G Impact of noise correlations.** **(A)** An example quadruplet of tuning curves. Solid lines represent spike count averages from data, shaded areas represent neuronal variability. **(B)** Sensitivities of quadruplet from panel A computed either taking into account noise correlations (blue) or neglecting them by shuffling the cells’ responses with respect to the trials. **(C)** Difference between correlated and shuffled quadruplet sensitivity. Noise correlations yield a moderate, but systematic improvement in encoding.(PDF)
