# Supplementary material for: Direction-selective retinal ganglion cells encode motion direction uniformly, despite having discretely distributed cardinal preferences
Source: PLoS Comput Biol. 2026 Nov 2;22(11):e1014872. doi: 10.1371/journal.pcbi.1014872 (PMC13739222; doi:10.1371/journal.pcbi.1014872)
Supplement: Supplementary file 1 [file pcbi.1014872.s001.pdf]

SUPPLEMENTARY INFORMATION

A. The Stimulus Specific Information offers a good measure for the stimulus sensitivity

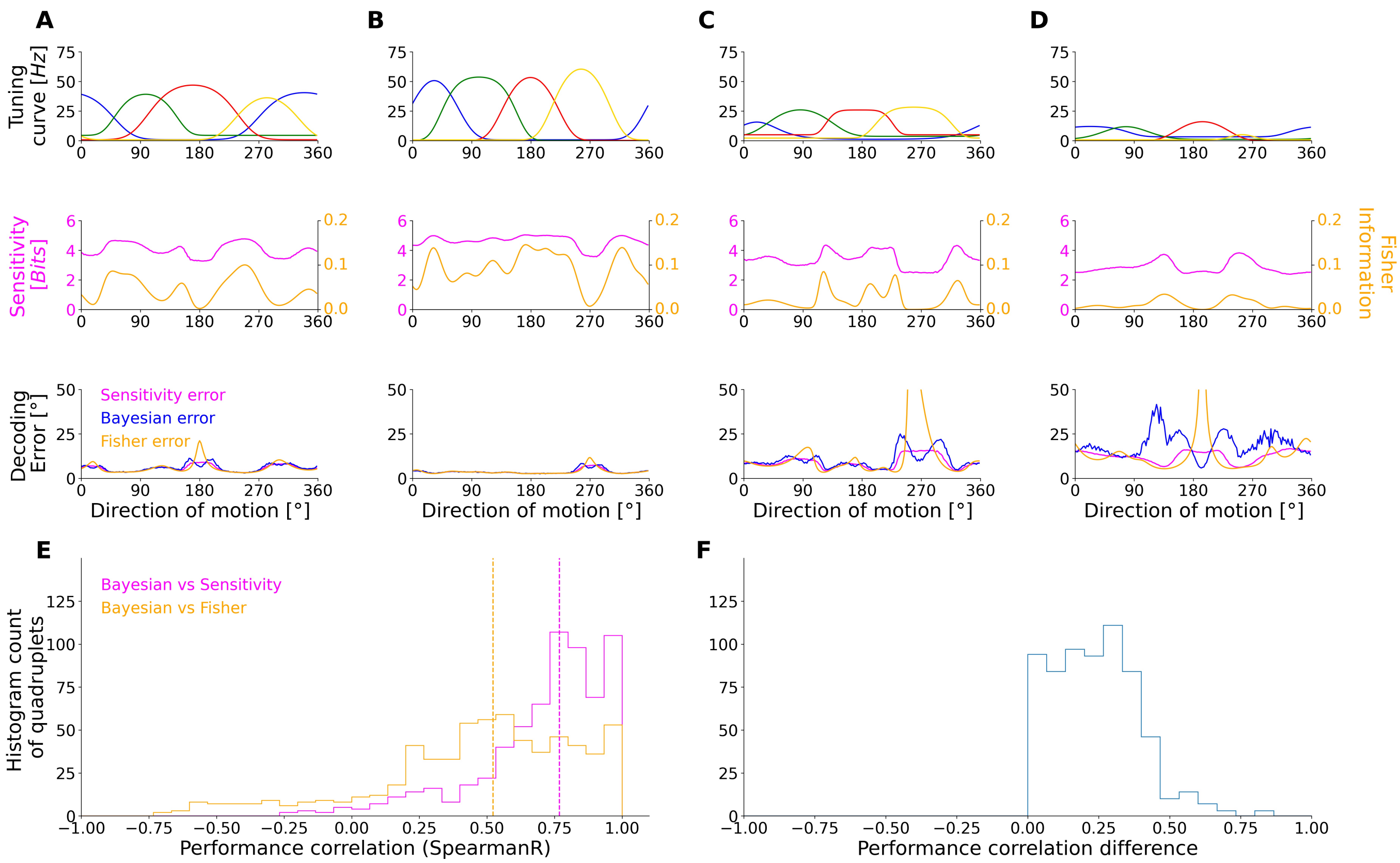

FIG. A. **The Stimulus Specific Information offers a good measure for the stimulus sensitivity.** **(A)** upper - Example quadruplet of tuning curves from Fig 1A; middle - Population sensitivity of Fig 1A quadruplet in magenta and its Fisher information $FI(\theta)$ in orange; bottom - Decoding errors estimated from the sensitivity, Fisher Information or a Bayesian unbiased decoder. The sensitivity error is proportional to $e^{-SSI(\theta)}$ with the SSI expressed in $nats$. The Fisher error is obtained from the unbiased Cramér-Rao bound as $^1/\sqrt{FI(\theta)}$, the inverse square root of the Fisher information. We assume a flat prior $p(\theta) = {}^1/|\Theta|$. Notice the Fisher information peaking around 180°, in correspondence to the red cell from the uppermost panel. This is due to the slopes of all 4 tuning curves being vanishingly small in that area, resulting in a Fisher Information error that tends to extremely large values where the Bayesian decoder error is bounded. Because the Fisher information is small, the decoding bias is large [43, 44] and the unbiased Cramér-Rao bound is violated. **(B)** Same as in A, but for Fig 1B. **(C,D)** Some quadruplets display a Fisher information that, due to their particular configuration of tuning curve slopes, tend to zero in certain points. Consequently the associated decoding error estimates grow to values which egregiously exceed the Bayesian decoding error. **(E)** Spearman Rank correlation coefficients between the Bayesian decoding error and either $e^{-SSI(\theta)}$ (magenta) or $^1/\sqrt{FI(\theta)}$ (orange). We can see that for $N = 648$ quadruplets the Sensitivity error estimate correlates with the Bayesian error more than the Fisher information error does. **(F)** Difference between Sensitivity and Fisher performance in terms of Spearman Rank coefficients of correlation to the Bayesian decoding error. The differences are done quadruplet-by-quadruplet, showing that for all of them the Sensitivity performance correlates with the Bayesian error more than the Fisher performance does.

A system which is highly sensitive to a particular stimulus should be expected to yield a small decoding error when probed for that stimulus. The Stimulus specific information (SSI) measures the average information carried by responses conditioned on stimuli that evoked them, so we should expect it to bear some relation to the decoding error. More precisely, upon stimulation a system generates a response sampled from a likelihood distribution conditional on the specific stimulus presented ($p(\mathbf{r}|\theta)$); this response should "point" to the stimulus which causes it, and the degree to which a one-to-one mapping can be established corresponds to the sensitivity of the system to the stimulus in question.

In order to verify that the SSI relates to the decoding error, and is therefore a good measure of sensitivity, we need

to link it with the error of a Bayesian decoder. The error of a Bayesian decoder is often defined as:

$$\sigma^2_{dec}(\theta) = \sum_{\mathbf{r}\in\mathbf{R}} p(\mathbf{r}|\theta)\cdot\left(\hat{\theta}(\mathbf{r})-\theta\right)^2 \tag{6}$$

However decoding error can also be measured with the average variance of the posterior:

$$\sigma^2_{post}(\theta) = \sum_{\mathbf{r}\in\mathbf{R}} p(\mathbf{r}|\theta)\cdot\sum_{\theta'\in\Theta} p(\theta'|\mathbf{r})\left(\hat{\theta}(\mathbf{r})-\theta'\right)^2 \tag{7}$$

where $\hat{\theta}(\mathbf{r})$ is the average of the posterior $p(\theta|\mathbf{r})$. Note that $\sigma^2_{dec}(\theta)$ and $\sigma^2_{post}(\theta)$ have the same mean: $\langle\sigma^2_{dec}(\theta)\rangle_{p(\theta)} = \langle\sigma^2_{post}(\theta)\rangle_{p(\theta)}$. Up to higher order corrections, and provided that the posterior $p(\theta|\mathbf{r})$ has a small enough variance, we can ignore the fact that the stimulus ensemble is bounded, and use the fact that the Gaussian distribution maximizes the entropy of a random variable. We can then obtain an approximate lower bound of the average posterior variance in terms of the SSI:

$$\sigma^2_{dec}(\theta) \approx \sigma^2_{post}(\theta) \geq \sigma^2_{SSI}(\theta) \equiv \frac{|\Theta|^2}{2\pi e} e^{-2\cdot SSI(\theta)} \ , \tag{8}$$

Eq 8 shows that the SSI can provide an estimate of the decoding error, and so can be used to quantify the stimulus sensitivity.

The Fisher information is another quantity from information theory that can be linked with decoding error via the Cramér-Rao bound [45]. For our system, neglecting noise correlations and therefore supposing conditionally independent cell responses, the Fisher information takes on a simple form dependent only on the properties of the tuning curves constituting the quadruplet:

$$FI(\theta) = \sum_{k=1}^{4} \frac{\left(f'_{TC_k}(\theta)\right)^2}{f_{TC_k}(\theta)} = \sum_{k=1}^{4} FI_k(\theta) \tag{9}$$

Simplicity and relatively direct interpretability make the Fisher information an often-used measure of performance in neuroscience [42, 44, 46]. That utility notwithstanding, the Fisher information presents a striking vulnerability in the specific case of small populations with tuning curves that can flatten in extended regions of the stimulus ensemble, such as the Flat-Topped Von Mises. In such a case, the probability that all the tuning curve slopes (almost) equal zero is non-negligible (Figs A(A-D), top panels). Consequently, the Fisher information will take on small values in those regions (Figs A(A-D), middle panels), which will in turn lead to large values of its inverse - the Fisher information decoding error estimate from the Cramér-Rao bound (Figs A(A-D), bottom panels). This would lead to the implausible conclusion that the region is uninformative, even if there a cell is spiking maximally.

Figs A(A-E) show that $\sigma^2_{SSI}(\theta)$, from Eq 8, correlates with the Bayesian decoding error more strongly than does the Fisher error. The medians of the histograms are respectively 0.77 and 0.52, while the $mean \pm std$ are, again respectively, 0.71 ± 0.24 and 0.47 ± 0.37. Furthermore, there is a substantial number of quadruplets for which the Fisher error negatively correlates with the Bayesian error. On a quadruplet-by-quadruplet basis the sensitivity error more closely correlates with the Bayesian decoding error compared to its Fisher information counterpart (Fig A(F)).

In summary, the Fisher information is a commonly used and often powerful tool for estimating a lower bound on the decoding error, and could in principle provide a robust measure of sensitivity. However in specific regions where the tuning curves flatten, the Fisher information fails to provide a plausible decoding error estimate. On the other hand, the sensitivity error based on SSI does not suffer from such a drawback. Instead it closely approximates the Bayesian decoding in the small noise limit. Finally, its high correlation with the Bayesian decoding error is a property which one should hope to find in an appropriately defined measure of sensitivity.

B. Impact of receptive field proximity on sensitivity of On-Off DS RGCs

The receptive fields of On-Off DS RGCs tile the rabbit retina with minimal same-type overlap, implying that a small local stimulus is processed by one cell of each subtype. This motivated the choice of quadruplets of cells as processing units. These quadruplets were generated without regard for the proximity of the cells' receptive fields. However, it is of interest to study the impact of receptive field proximity on the tuning of neighboring cells, and therefore their sensitivity.

To address this point, we partitioned our population of quadruplets into "proximal" and "non-proximal" groups. Proximal quadruplets were defined as those composed of cells whose centers of mass were all within $300\mu m$ of one

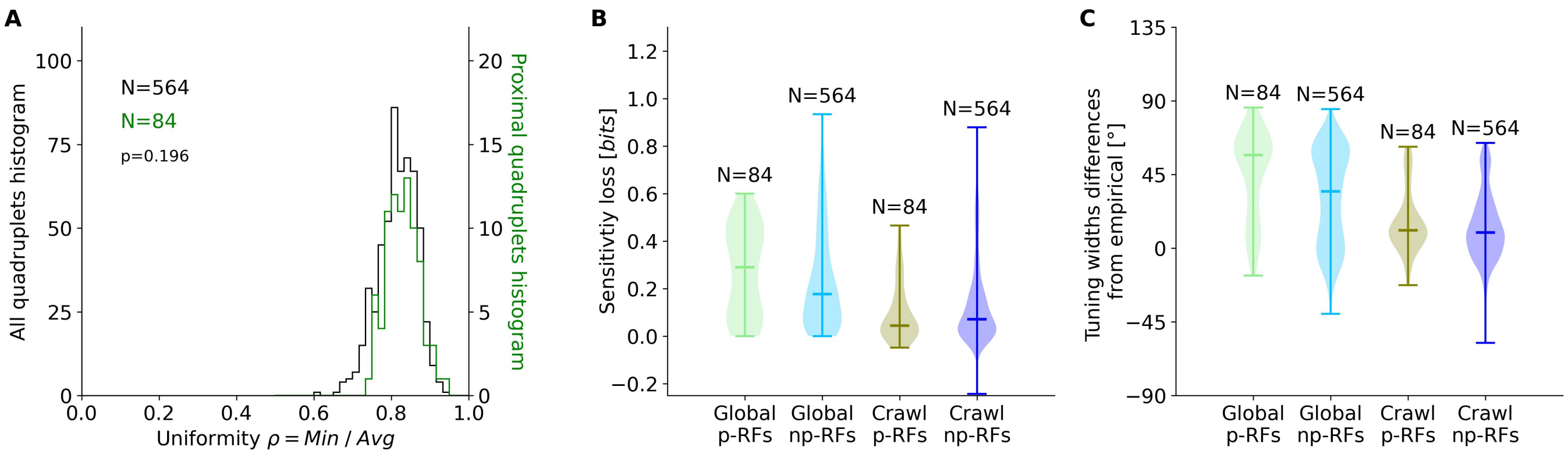

FIG. B. **Impact of receptive field proximity on sensitivity of On-Off DS RGCs.**
(A) Uniformity distributions for quadruplet subpopulations with non-proximal (np-RFs, black) and proximal receptive fields (p-RFs, green). **(B)** Violin plots of minimum sensitivity losses for empirical quadruplets relative to global (light) and crawl (dark) optima. Proximal quadruplets show larger losses at empirical average tuning widths compared to non-proximal quadruplets, whereas crawl optima converge near empirical performance regardless of receptive field position. **(C)** Violin plots of width differences for global and crawl optima, separated by receptive field overlap. Global optima for proximal quadruplets favor narrow tuning widths, while non-proximal quadruplets display a more bimodal distribution; crawl optima remain close to empirical widths in both cases.

another. Out of 648 randomly generated quadruplets, 84 satisfied this criterion. Panel A shows the distribution of uniformity indices (defined as the ratio between minimum and average sensitivity) for proximal (green) and non-proximal (black) quadruplets. The two distributions exhibit substantial overlap. A Kolmogorov-Smirnov test yields a p-value of 0.195 (> 0.05), indicating that we cannot reject the null hypothesis that the two samples are drawn from the same underlying distribution. Within the limits of our dataset, this suggests that receptive field overlap does not have a significant impact on sensitivity uniformity.

We further compared the dependence of minimum sensitivity on tuning curve width for proximal and non-proximal quadruplets. Panel B shows violin plots of the minimum sensitivity losses of empirical quadruplets relative to the global (light colors) and crawl (dark colors) optima. The global optima of quadruplets fall at both narrow widths and those close to the empirical widths, as expected. Furthermore, the crawl algorithm converges to performances close to the empirical values, irrespective of receptive field position. This means that both proximal and non-proximal quadruplets indeed exhibit minimum sensitivity peaks at values similar to the performances achieved by the empirical quadruplets.

Panel C shows violin plots of width differences for the global and crawl optima of minimum sensitivity, separated by receptive field overlap. The global optima of proximal quadruplets are predominantly located at narrow average tuning curve widths, whereas non-proximal quadruplets display a more pronounced bimodal distribution. Nevertheless, the crawl algorithm converges to widths close to the empirical ones regardless of receptive field placement.

These results suggest that receptive field proximity has, at most, a limited influence on the minimum-sensitivity landscape. Both proximal and non-proximal quadruplets exhibited qualitatively similar behavior. Some differences between the distributions of optimal widths and performances were observed in the global optima, which may indicate a modest effect of receptive field position on sensitivity. However, we cannot firmly conclude that. The number of spatially proximal quadruplets was substantially smaller than in the full dataset (84 versus 564), limiting statistical power. In addition, individual cells were reused across multiple quadruplets, introducing correlations between samples and reducing the effective sample size. Consequently, while the data allow for a possible influence of receptive field proximity on minimum sensitivity, they do not provide sufficient evidence to draw firm conclusions regarding its role.

C. Effect of prior probability

Throughout this work, we used a uniform prior over the stimulus ensemble. However, real-world stimuli may exhibit non-uniform prior probabilities over direction of motion, for example by the optic flow over the retina [35]. It is therefore of interest to study whether our results would remain applicable in the latter case.

To address this point, we considered a simplified geometric model in which preferred directions vary systematically with eccentricity due to optic flow constraints. In this framework, each direction is expressed as a linear combination of "base vectors" tangent to ellipses defined by the naso-temporal and dorso-ventral axes, with magnitudes

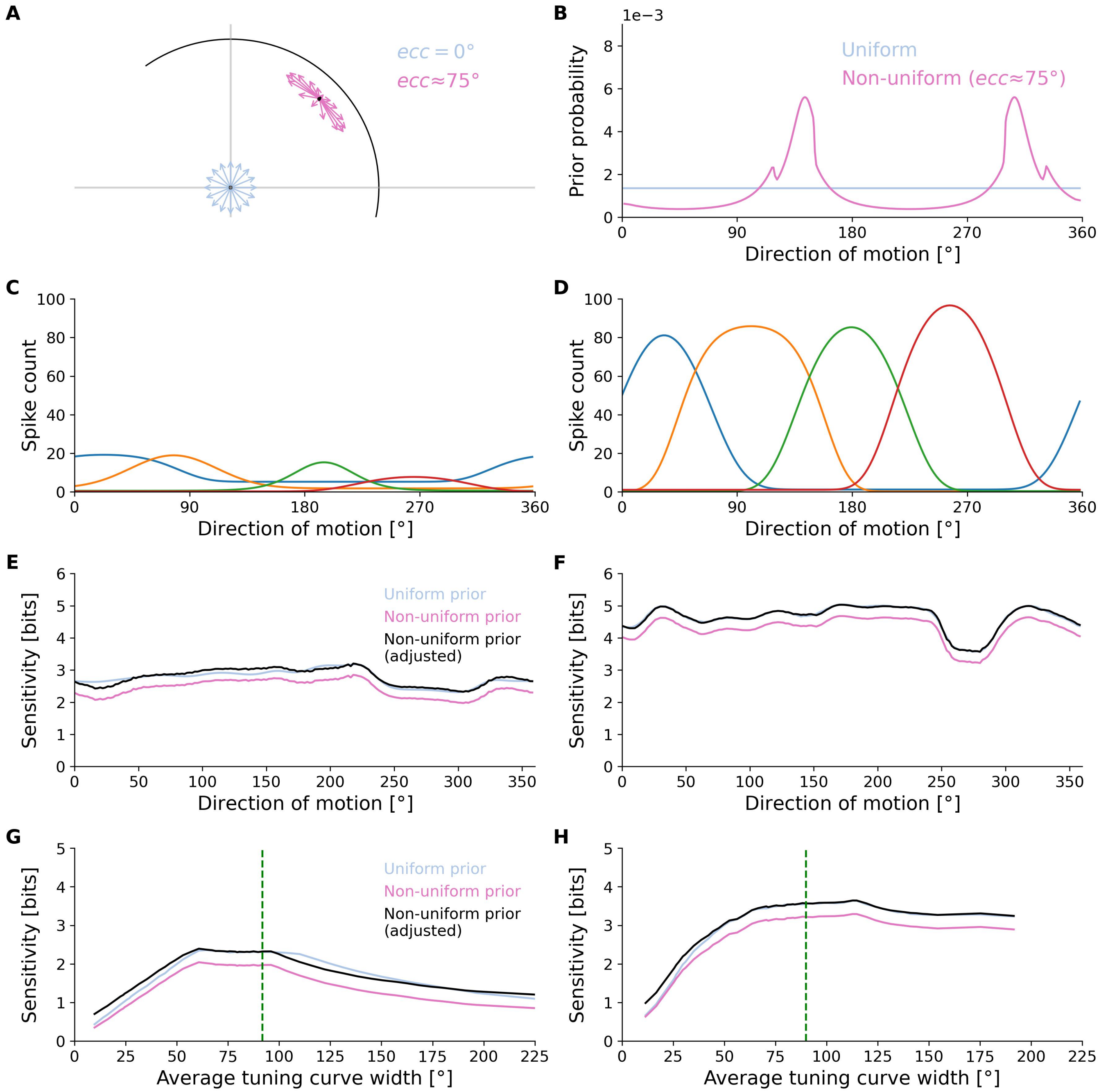

FIG. C. **Effect of prior probability.**
(A) Simple geometric model of how the probability distribution of directions of motion changes with eccentricity. The arc represents a quadrant of the retina, the blue arrows represent the distribution of directions of motion in the retina's geometric center (equiprobability is represented by identical arrow length), while the pink arrows represent the modified non-uniform prior at an eccentricity of $\approx 75^\circ$. **(B)** The uniform and non-uniform priors obtained through the model in panel A, shown in blue and pink, respectively. **(C,D)** The quadruplets with the lowest and highest average spike counts, respectively, from our population of quadruplets. **(E,F)** The sensitivities of the weakest and strongest quadruplets, computed using the uniform prior (blue) and the non-uniform prior (pink). The adjusted sensitivity (black) is obtained by shifting the pink curve by the difference between the uniform and non-uniform prior entropies, to highlight the overlap with the uniform prior sensitivity and the contribution of the likelihood in its computation. **(G,H)** The minimum sensitivities as a function of average tuning curve width (obtained as in Fig 3 of the main text), computed using the uniform and non-uniform priors. The black lines are obtained using the non-uniform prior and then adjusted as in panels E and F.

determined by the orthogonal projection of a hemispherical surface onto the retinal plane. As eccentricity increases, this construction produces progressively larger deviations from equidistant direction configurations, consistent with experimental observations.

We assumed that the prior distribution over motion directions is uniform at the geometric center of the retina (panel A, blue arrows), and becomes non-uniform with increasing eccentricity (panel A, pink arrows). The corresponding priors are shown in Fig C(B). Notably, at an eccentricity of approximately 75°, the prior becomes strongly bimodal. To probe the interaction between prior and likelihood, we focused on two representative quadruplets corresponding to extreme signal-to-noise regimes: one with low average spike count maxima (Fig C(C)) and one with high maxima (Fig C(D)). This choice reflects the dependence of our sensitivity measure on SNR ratio, and allows us to disentangle the contribution of the prior from that of the likelihood.

The resulting sensitivities are shown in panels E and F. The blue curves correspond to the sensitivity computed under a uniform prior Π_U , $I_{SSI}(\Pi_U\ ;\ \theta)$, while the pink curves correspond to the sensitivity under a non-uniform prior Π_{NU} , $I_{SSI}(\Pi_{NU}\ ;\ \theta)$. The dominant difference between these curves is a constant offset, which can be attributed to the difference in prior entropies: $\varepsilon = H[\Pi_U] - H[\Pi_{NU}]$. To account for this, we define an adjusted sensitivity, $I_{SSI}(\Pi_{NU}\ ;\ \theta) = I_{SSI}(\Pi_U\ ;\ \theta) + \varepsilon$ (shown in black), which largely restores alignment with the uniform-prior case.

We also examined how the minimum sensitivity landscape depends on the prior by repeating the analysis of Fig 3 in the manuscript under both priors. Figs C(G) and C(H) in show the results for the two representative quadruplets. In both cases, the qualitative conclusion remains unchanged: the tuning configurations lie close to those that maximize minimum sensitivity, with only a small deviation from the global optimum.

Interestingly, for the high-firing-rate quadruplet (Fig C(D)), the sensitivity (Fig C(F)) and minimum-sensitivity curves (Fig C(H)) are nearly identical, differing primarily by a systematic offset attributable to the different prior entropies, as reflected in the near-complete overlap of the black and blue curves. This indicates that, in this regime, the shape of the sensitivity profile is largely determined by the likelihood. For the low firing-rate quadruplet, a small deviation is apparent, although its magnitude remains modest and does not alter the overall conclusions. This supports the view that, in the system considered here, prior-induced effects are secondary to those arising from the likelihood, particularly in regimes of high SNR ratio.

D. Sensitivity of populations composed of 8 and 16 On-Off DS RGC

To clarify the relationship between population size, uniformity of sensitivity and the MaxMin principle, we randomly generated 200 8-cell and 200 16-cell populations from experimentally measured tuning curves. Due to the limited number of available cells per preferred direction per experiment, some cells might be taken twice for construction 8-plets or 16-plets. In each case, populations were constructed with a fixed number of cells per preferred direction (2 and 4, respectively). Fig D(A) shows an example 8-cell population (top), together with its corresponding sensitivity profile (bottom). Fig D(B) shows the analogous construction for a 16-cell population. As population size increases, sensitivity minima become less pronounced, consistent with improved tiling of the stimulus space by the tuning curves. In addition, because more cells contribute to encoding each stimulus, the overall (average) sensitivity increases.

Fig D(C) shows that uniformity remains high for both 8- and 16-cell populations, with a reduced fraction of low-uniformity cases compared to 4-cell populations. The median uniformity indices for 4-, 8-, and 16-cell populations are quite compatible: 0.82, 0.82, and 0.83, respectively.

We next examined the distributions of performance losses relative to globally optimal minimum- and average-sensitivity solutions, as a function of average tuning curve width, and compared these to the performance of populations constructed from empirical tuning curves. Minimum- and average-sensitivity results are shown in blue and red, respectively. For reference, the first two violin plots in Fig D(D) reproduce the corresponding histograms from Fig 3D of the main text. As in the case of quadruplets, 8- and 16-cell populations exhibit smaller losses relative to the globally optimal minimum sensitivity than relative to the globally optimal average sensitivity. Notably, as population size increases, the global optimum of average sensitivity increases, leading to a larger loss relative to empirical tuning curve widths under this objective. Nevertheless, across all population sizes, empirical configurations remain closer to the global optima under the minimum-sensitivity criterion (Fig D(D)).

This trend is further supported by the crawl algorithm (Fig D(E)), which preserves the magnitude of the loss relative to the optimal average-sensitivity solution, while converging toward configurations consistent with empirical minimum-sensitivity performance. Minor negative values in Fig D(E) arise from small numerical fluctuations near the minimum-sensitivity peak, with the exception of one quadruplet exhibiting a local maximum at very large widths; this local maximum is approximately 0.25 *bits* lower than the performance at empirical tuning curve widths.

With respect to tuning curve widths, 8- and 16-cell populations show global optima for minimum sensitivity both at relatively narrow widths (approximately 45° narrower than empirical values) and at widths close to empirical measurements, as shown in the blue distributions in Fig D(F). In contrast, global optima of average sensitivity consistently

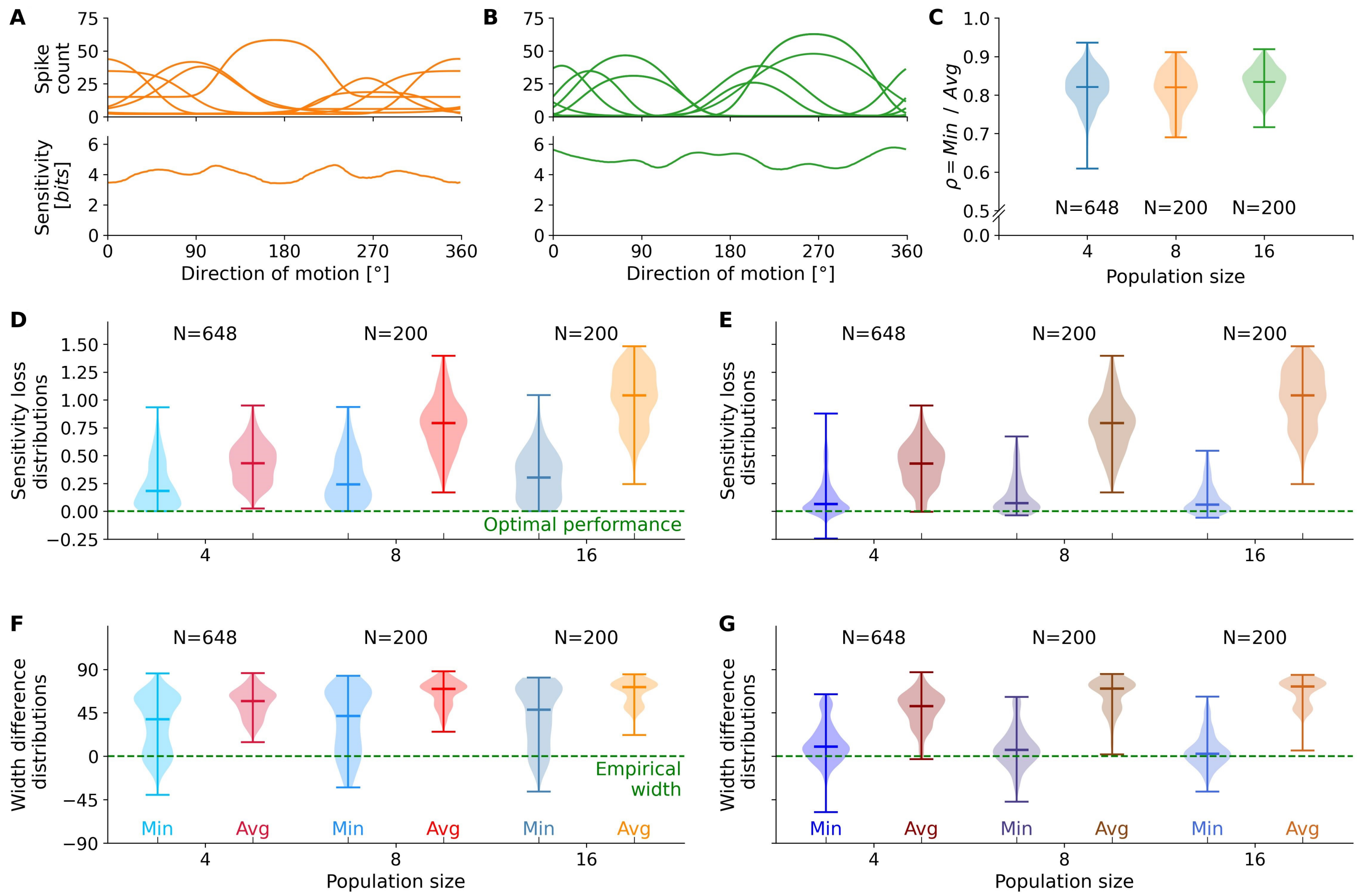

FIG. D. **Sensitivity of populations composed of 8 and 16 On-Off DS RGC.**
(A) Example 8-cell population: two neurons per preferred direction (upper) and corresponding sensitivity profile (lower). **(B)** As in panel A, but for a 16-cell population with four neurons per preferred direction. **(C)** Uniformity indices for 4-, 8-, and 16-cell populations (blue, orange, green). Variability in uniformity decreases with increasing population size. **(D)** Minimum (blue) and average (red) sensitivity losses relative to global optima. Empirical populations are closer to optimal under the minimum-sensitivity objective. **(E)** The crawl algorithm converges toward empirical configurations along the minimum-sensitivity landscape, while identifying non-empirical global optima under the average-sensitivity objective. Although average-sensitivity optima deviate further from empirical configurations, the crawl still recovers empirical minimum-sensitivity solutions. **(F)** Distributions of differences between empirical tuning widths and globally optimal widths for 8- and 16-cell populations mirror those of quadruplets. Narrow widths optimize average sensitivity, whereas minimum sensitivity is achieved across a broad range of widths, many close to empirical values. **(G)** The crawl algorithm recovers empirically consistent tuning widths along the minimum-sensitivity landscape for 4-, 8-, and 16-cell populations.

occur at narrow tuning widths across population sizes. However, when optimizing under the minimum-sensitivity objective, the crawl algorithm converges to empirically consistent tuning widths (Fig D(G)), while converging to distinct non-empirical optima under the average-sensitivity objective.

Together, these results further support the MaxMin principle as a candidate organizing rule for On-Off DS RGCs, and indicate that the crawl algorithm can be a viable principled route to recovering empirically observed tuning curve widths.

E. Sensitivity simulation for triplets of ON cells yields similar, albeit less marked, results as for quadruplets

Apart from On-Off Direction Selective Retinal Ganglion cells, another type of direction selective cell found in the rabbit retina is the On-type direction selective cell. Unlike On-Off cells, which respond to both increases and decreases in luminosity, On cells respond only to luminosity increase. Furthermore, this type feature 3 preferred directions [10, 14, 36].

As On DS cells recordings are difficult to obtain, we estimated the parameters of their tuning curves from [36].

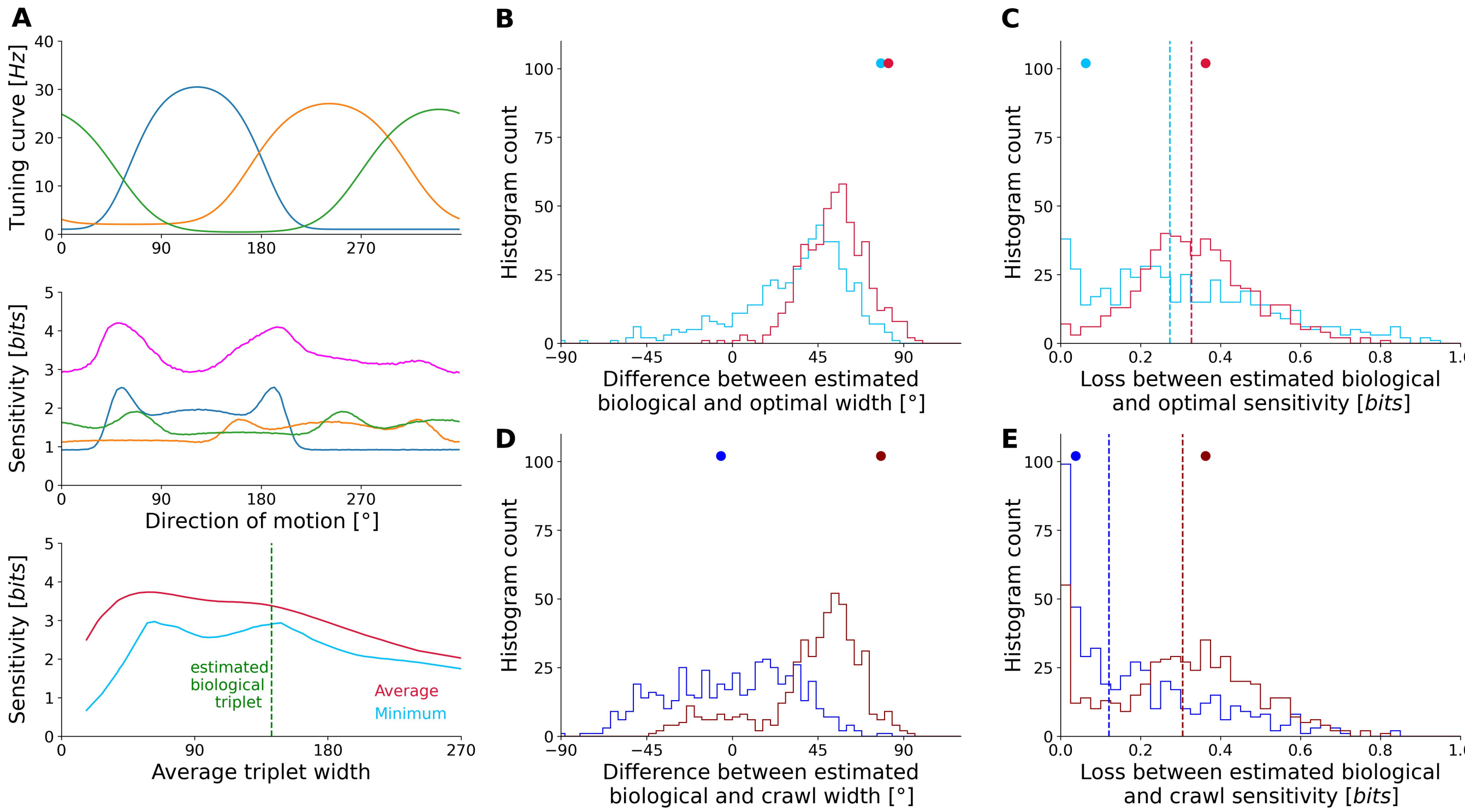

FIG. E. **Sensitivity simulation for triplets of ON cells yields similar, albeit less marked, results as for quadruplets.** **(A)** Upper: example of a triplet of tuning curves; middle: example of individual and population sensitivities associated to example triplet; Bottom: behavior of triplet sensitivity average and minimum (respectively lightred and lightblue) as function of simulated tuning curve widening. **(B)** Difference between the estimated average "biological" triplet width and the width associated to the global maximum of the average and minimum sensitivity (lightred and lightblue respectively), analogous to Fig 3C. There appears to be little difference between the average triplet widths that maximize the minimum sensitivity and the average sensitivity. Most of the maxima occur at tuning curve widths approximately 45° narrower than the estimated biological width. The colored markers identify the bins containing the example triplet from panel A. **(C)** Histograms of performance loss of estimated biological minimum and average sensitivity compared to the associated maxima (respectively lightblue and lightred), analogous to Fig 3D. The trend is similar to that of Fig 3D, but with a markedly smaller peak around zero of the minimum sensitivity histogram. **(D)** Difference between the estimated average "biological" triplet width and the width associated to the crawl-obtained maximum of the average and minimum sensitivity (darkred and darkblue respectively), analogous to Fig 4B. While the histogram associated to the average is largely the same as the on in Fig 4B, the histogram associated to the minimum, although centered in zero, is much wider than its quadruplet counterpart. **(E)** Histograms of performance loss of estimated biological minimum and average sensitivity compared to the associated crawl-obtained maxima, analogous to Fig 4C. As for quadruplets, the crawl-obtained widths favor maximizing the sensitivity minimum rather than the average, although the result is less marked in the case of cell triplets.

Estimated Von Mises concentration for On cells was approximately 1.15 times larger than for On-Off cells, and the average firing rate approximately 66% of an equivalent On-Off cell (Fig 2 in [36]). Each simulated experiment assigned cells to three groups to mimic three preferred directions and generated all unique triplets with one cell per group.

Tuning curve widening simulation results are shown in Fig E. On-cell tuning curves are less energetic and wider than On-Off cells (Fig E(A), upper panel), producing wider individual sensitivities with fewer prominent peaks (Fig E(A),middle panel). Compared to quadruplets, triplets show similar sensitivity minimum and average profiles as a function of average tuning curve width (Fig E(A), bottom panel).

Global maxima analysis indicates that optimal widths for both criteria are $\sim 45°$ narrower than the estimated biological widths (Fig E(B)). Loss histograms retain quadruplet trends, but neither minimum nor average can reliably be considered a relevant optimization criterion for these cells (Fig E(C)). Crawl procedure results show that optimal triplets' average widths are widely distributed around estimated biological widths, with lower performance loss for sensitivity minima versus averages. Initial parameter estimates were rough but trends and statistical behaviors persisted.

In conclusion, our analyses suggest that triplets of direction selective cells exhibit relatively uniform sensitivity and that the estimated biological tuning widths (which are believed to be broader for On DS RGCs than for On-Off DS

RGCs) are more consistent with optimization of the minimum sensitivity than of the average sensitivity. Similarly, the crawling algorithm tends to converge toward widths associated with high minimum sensitivity, as observed for quadruplets. However, because these analyses rely on estimated tuning widths rather than direct physiological recordings, the conclusions should be regarded as preliminary. While the results are consistent with the trends observed in quadruplets and point in the same direction as our proposed framework, additional experimental data will be required to determine how strongly these principles apply to On DS RGC populations.

F. Relationship between spike count average and variance for On–Off DS RGCs

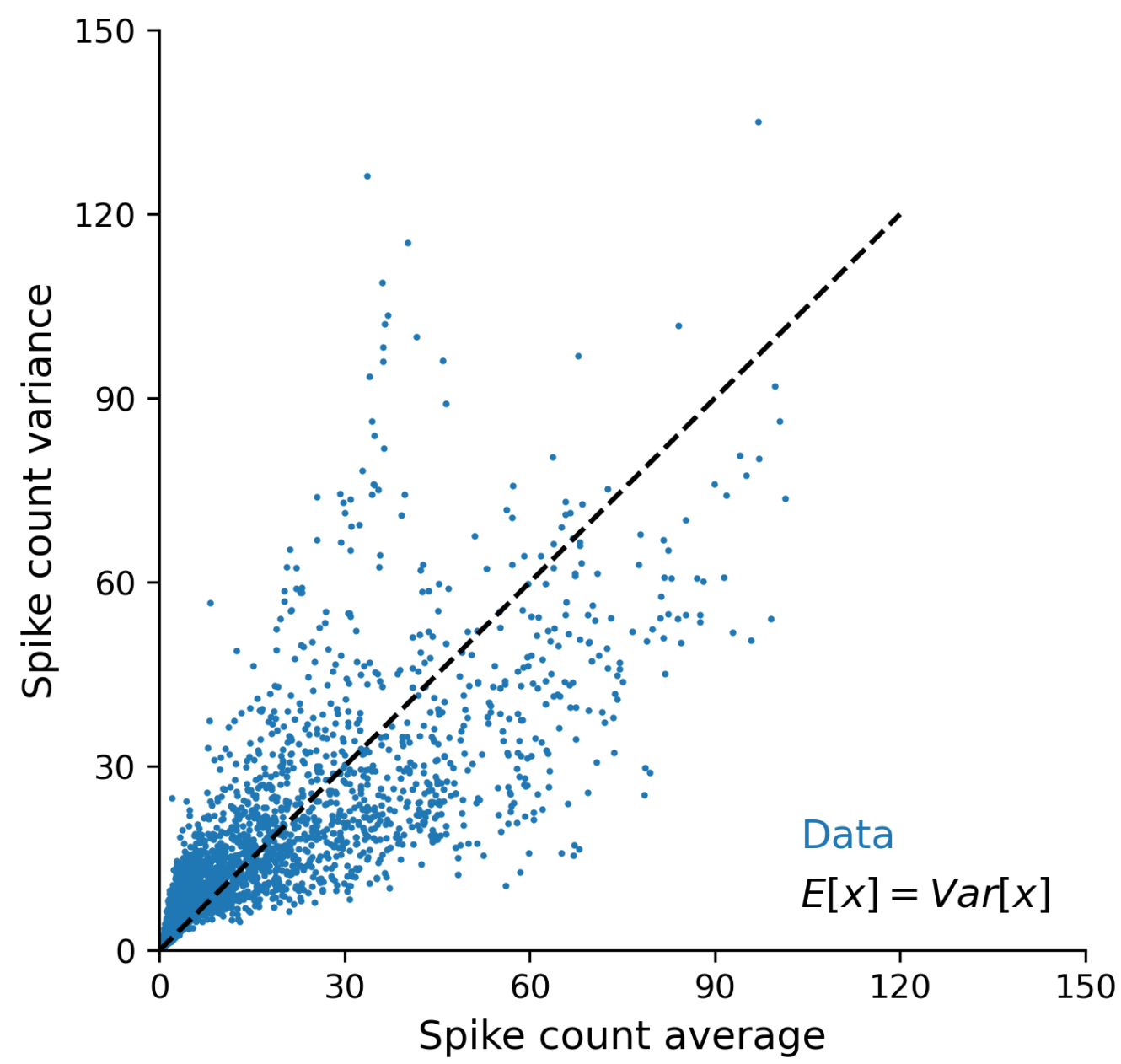

FIG. F. Each point represents the mean and variance of a cell's spike count when presented with some direction of motion. The plot contains data from $N = 90$ cells and $|\Theta| = 36$ directions. The dashed line indicates Poisson variability ($E[x] = \mathrm{Var}[x]$). The data follow the Poisson prediction for low to moderate spike counts, but show increasing dispersion for larger mean spike counts. Nevertheless, the dispersion is approximately symmetric with respect to the identity line, suggesting that assuming an over- or under-dispersed variability model would not substantially alter our conclusions. Data plotted from [22, 23].

In this work we assumed Poisson noise, whose Fano factor equals one. For low and moderate spike counts this approximation works well, as can be seen from Fig F. At high spike counts, variability increased but remained roughly symmetric around the identity line, with some cells sub-Poisson and others super-Poisson. The lack of a clear trend suggests that refining the noise model would likely not substantially improve accuracy.

G. Impact of noise correlations

We considered conditionally independent populations of neurons, that is populations of neurons whose response likelihoods can be factorized as a product of the single cell likelihoods:

$$p(r_1, ..., r_N|\theta) = \prod_{i=1}^{N} p(r_i|\theta) \tag{10}$$

This was in many cases necessary in order to reduce computational and time complexity. However, a biologically more accurate model of population response likelihood would have to account for noise correlations as well. The data we worked with was analyzed in and obtained from Franke et al. [23], whose study showed that noise correlations between neurons indeed exist and improve coding. The SSI is able to capture this improvement as well (panels B and C of Fig G). One notable difficulty inherent in the "noise correlations extension" of our work would be modeling

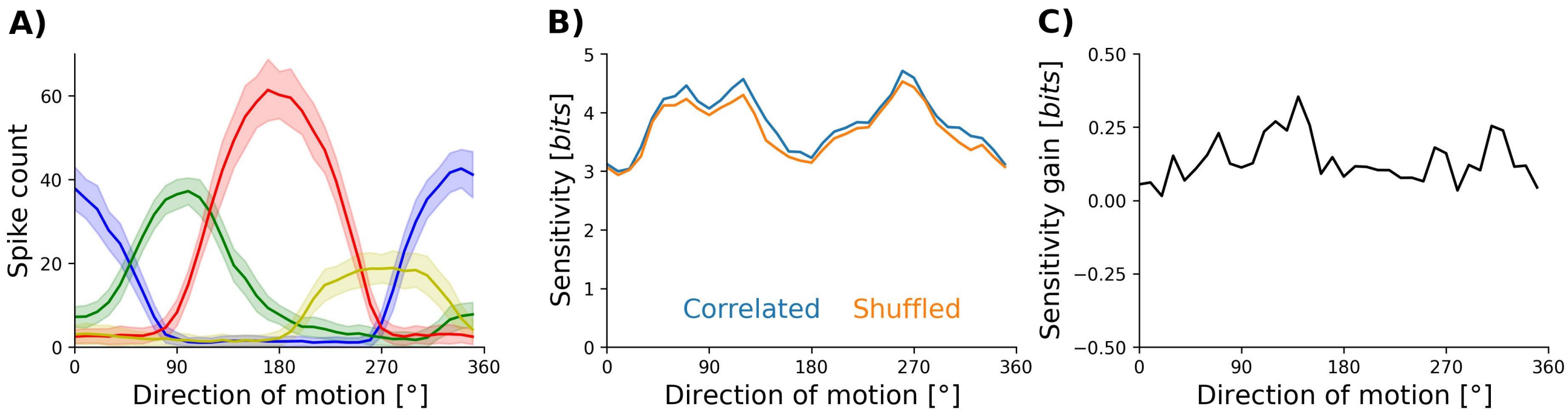

FIG. G. **(A)** An example quadruplet of tuning curves. Solid lines represent spike count averages from data, shaded areas represent neuronal variability. **(B** Sensitivities of quadruplet from panel A computed either taking into account noise correlations (blue) or neglecting them by shuffling the cells' responses with respect to the trials. **(C)** Difference between correlated and shuffled quadruplet sensitivity obtained by subtracting the "shuffled sensitivity" from the "correlated sensitivity". Noise correlations yield a moderate, but systematic improvement in encoding.

the correlations for tuning curves with modified widths. We did not pursue this line of inquiry because, although we observed the same positive effect on coding reported by Franke et al., a preliminary analysis including noise correlations did not alter our conclusion that quadruplet sensitivity is homogeneous.
