# Supplementary material for: Direction-selective retinal ganglion cells encode motion direction uniformly, despite having discretely distributed cardinal preferences
Source: PLoS Comput Biol. 2026 Nov 2;22(11):e1014872. doi: 10.1371/journal.pcbi.1014872 (PMC13739222; doi:10.1371/journal.pcbi.1014872)
Supplement: Supplementary file 2 [file pcbi.1014872.s002.pdf]

Authors' response and summary of changes

Dear Editor and reviewers,

We appreciate your role in facilitating the review process of our manuscript. We would also like to thank the reviewers for their thorough evaluations and insightful comments, which have substantially helped us improve our manuscript.

Before replying to your comments in detail, we would like to give you a synthetic overview of the major changes in the manuscript following your suggestions and comments:

1. We have added an entire new section E containing an analysis of data with a different bar speed, that was not included in the former version of the manuscript.
2. We have added four supplementary sections, with accompanying figures on (i) Impact of receptive field proximity; (ii) Impact of non-uniform prior; (iii) Sensitivity of populations with 8 or 16 DS RGCs; (iv) Sensitivity of On DS RGCs

This additional work and the PhD defense of the first author are the main reasons beyond the long time it took us to prepare this rebuttal. We deeply apologize for this.

In the following, we address all comments and concerns from the reviewers in detail. Please note that the referees' comments are written in ***italic black***, our answers in **blue**, text referring to the old version of the manuscript in **green**, and text newly introduced in the manuscript in **red**. Corresponding changes in the manuscript are also highlighted in **red**.

Sincerely yours,
The authors

Reviewer #1:

This manuscript presents an elegant and conceptually insightful analysis of how tuning curves can be organized to ensure homogeneous coverage of all movement directions with a limited number of channels coding for motion direction. The methodological approach is rigorous, and the use of stimulus-specific information (as defined in Butts 2003) is well justified. Comparing minimum sensitivity (minimum stimulus-specific information) with average sensitivity (mutual information) provides a clear and effective way to assess both the presence and severity of blind spots. This analytical framework has potential relevance for a broad range of problems in computational biology and normative coding theories.

A key strength of the study lies in demonstrating that, while the average sensitivity curve yields a single optimum, the minimum sensitivity curve may display one or two "optimal" tuning widths. The "crawling" approach, in which tuning curves evolve from uniform to locally optimal configurations, offers an elegant explanation for why tuning curves are typically broad in biological systems, trading off maximum sensitivity for robustness.

We thank the reviewer for their careful reading of our paper and for their positive comments.

To further strengthen the manuscript and clarify the mechanisms shaping the sensitivity curves, the following points merit attention:

Major comments

1. Receptive field overlap:

It is not clear whether the quadruplets analyzed have similar receptive field locations and whether this influences the occurrence of one versus two peaks, i.e., do quadruplets with overlapping receptive fields consistently show a single peak or two peaks?

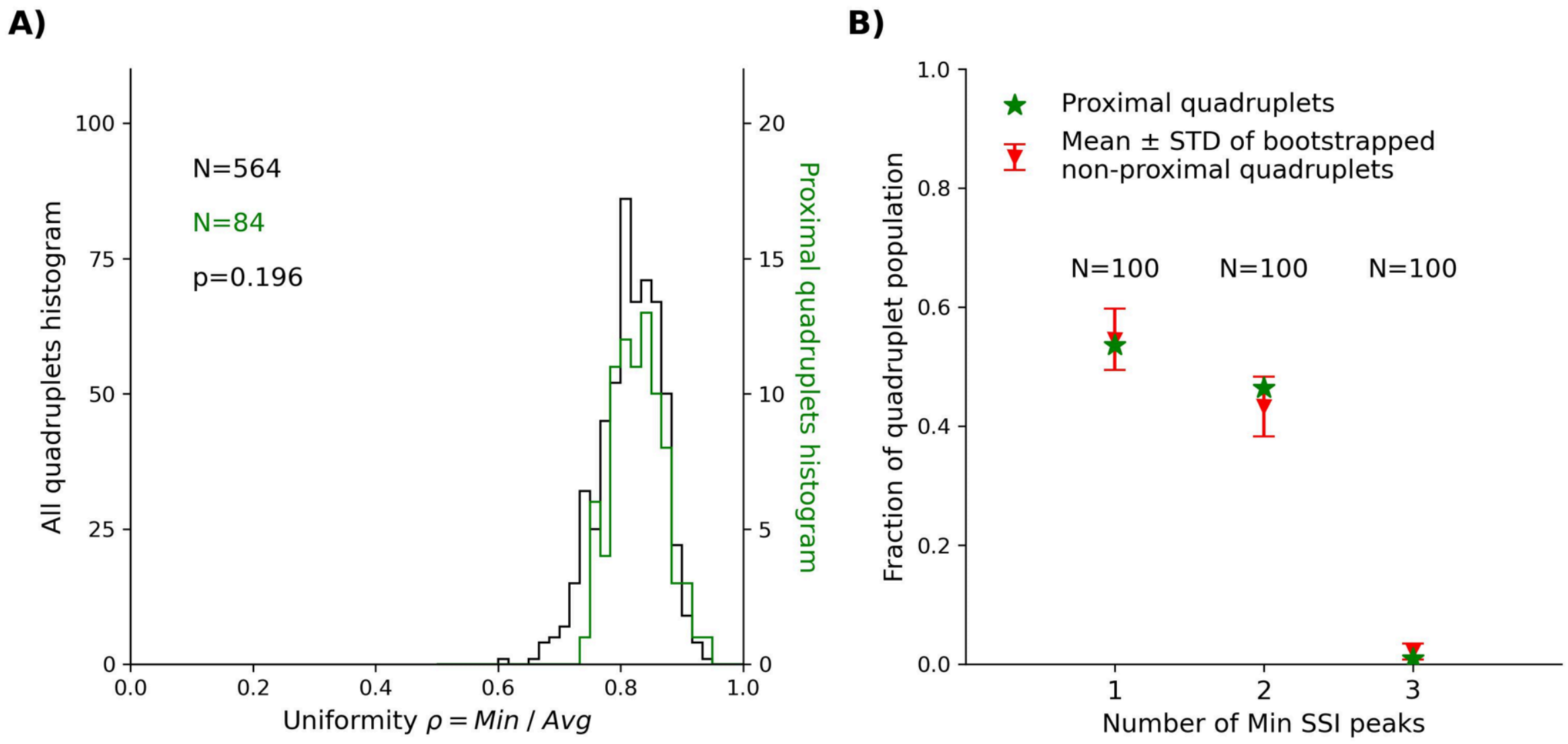

Figure 1: Receptive field overlap does not significantly impact the uniformity of quadruplet sensitivity or the number of minimum sensitivity peaks.

In brief, our analyses do not provide evidence supporting a relationship between receptive field location and number of minimum sensitivity peaks.

In order to study the relationship between RF location and number of minimum sensitivity peaks, we compared the sensitivity uniformity of the full population of randomly assembled empirical quadruplets with that of a subset composed of cells whose receptive fields were spatially proximal (defined as a center-of-mass distance < 300 μm). As shown in Panel A, the distributions of uniformity index values for these two groups are highly similar. A Kolmogorov - Smirnov test yielded p = 0.337, indicating that we cannot reject the null hypothesis that both samples are drawn from the same distribution.

We further examined whether spatial proximity influences the number of peaks in the minimum sensitivity curve as a function of average tuning curve width. Among proximal quadruplets (N = 84), 54% exhibited a single peak and 46% exhibited two peaks (Panel B, green markers). To provide a fair comparison, we repeatedly sampled (100 iterations) sets of 84 quadruplets from the larger pool of non-proximal quadruplets (N = 564) and computed the corresponding peak statistics. This analysis revealed no significant difference between proximal and non-proximal groups. This is evidenced by the fact that, for each number of minimum sensitivity peaks, the fraction of biologically proximal quadruplets falls within a

standard deviation from the fraction of non-proximal quadruplets exhibiting the same number of peaks.

We have included part of this analysis in the new supplementary section B and reported it in the discussion. See also our response to question no. 4 of reviewer 2

2. Non-equidistant spacing of preferred directions:

In the mouse retina, the spacing of preferred directions within a quadruplet is nearly equidistant in the central retina but not in the periphery (Sabbah et al., 2017, Nature). It would be informative to investigate whether such non-equidistant spacing alters the minimum sensitivity landscape. For example, is a single peak more likely in quadruplets with non-equidistant spacing, and do wider tuning curves become optimal under these conditions?

As the reviewer correctly pointed out, the relative configuration of preferred directions changes with eccentricity (according to Sabbah et al., *Nature*, 2017). We acknowledge the importance of studying the impact of these changes to our theory, insofar as concerns both the uniformity of sensitivity and the minimum sensitivity as a function of tuning curve width.

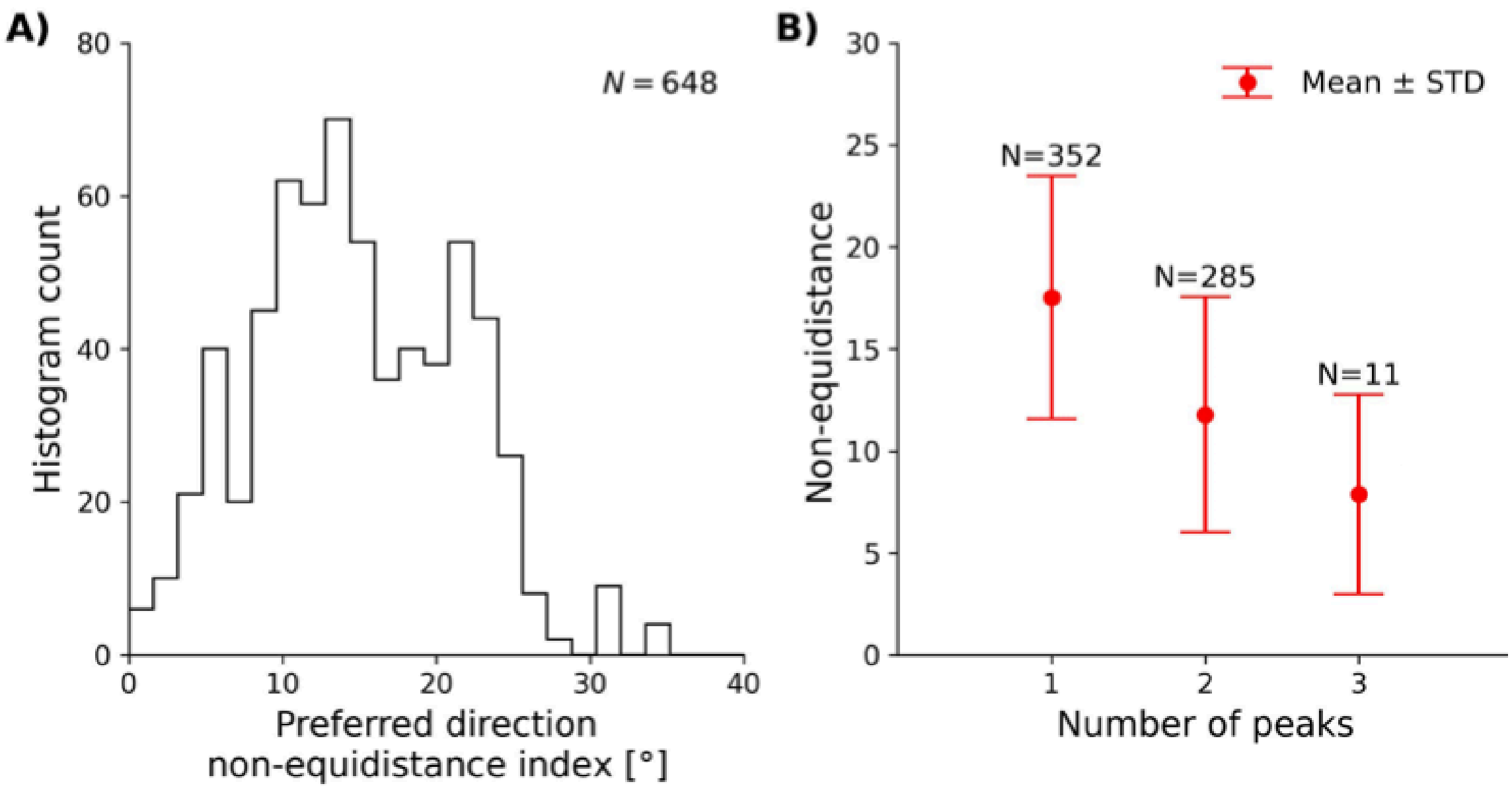

Figure 2: Non-equidistance of preferred directions (difference from perfect orthogonality) does not significantly impact the number of minimum sensitivity peaks.

We address the reviewer's question in two parts, first studying the relation between the number of minimum sensitivity peaks and non-equidistance of preferred directions (Figure 2), subsequently studying the impact of eccentricity on quadruplet sensitivity (Figure 3).

We first ask whether single peaks are more likely when preferred directions deviate from an equidistant configuration (i.e., 90° spacing). To do that, we define a non-equidistance index as the mean squared deviation between preferred directions of an empirical quadruplet and the equidistant reference configuration with the smallest deviation from the empirical deviations. Using this measure, we computed a non-equidistance index for each of the N = 648 quadruplets (Fig. 2A).

We then examined how this index relates to the number of peaks in the minimum sensitivity function for each quadruplet. As illustrated in panel B, there is a tendency for the number of peaks to increase as configurations approach equidistance (i.e., lower non-equidistance index), reinforcing the reviewer's intuition. However, due to the lack of data, this trend can not be statistically tested. This is due to the correlation between

non-equidistant indices of quadruplets containing the same cells (e.g. 2 or 3 out of the 4 cells repeated in multiple quadruplets). Conversely, it is not possible to perform robust statistical analyses by constructing quadruplets without repetition, as for certain preferred directions in certain experiments we have only one cell.

To further address the reviewer’s question, we performed simulations in which preferred directions were systematically varied in a manner consistent with the organization reported by Sabbah et al., while keeping all other tuning curve parameters fixed. Specifically, we implemented a simple geometric model relating eccentricity to preferred direction configuration (Fig. 3A), in which preferred directions align with tangents to ellipses defined by naso-temporal and dorso-ventral axes. As eccentricity increases, this model produces systematic deviations from equidistance, consistent with experimental observations.

For each eccentricity, we computed the uniformity index (defined as the ratio of minimum to average sensitivity) for all quadruplets (Fig. 3B, insets). Notably, the empirical tuning curves exhibit an approximately constant uniformity index across eccentricities. This suggests that, within this simple model of flow-induced preferred-direction deformation, eccentricity from the geometric center of the retina (and thus the degree of preferred-direction deformation) has little effect on sensitivity uniformity.

We then investigated how minimum and average sensitivity depend on average tuning curve width under these varying preferred direction configurations (Fig. 3, panels C and D). Starting from cardinal configurations ([0°, 90°, 180°, 270°]), we computed sensitivity curves as a function of tuning width and repeated the analysis for progressively more eccentric, and thus more non-equidistant configurations. As the reviewer insightfully anticipated, altering preferred directions reshapes the sensitivity landscape. In particular, configurations with equidistant preferred directions exhibit two local maxima in minimum sensitivity, whereas increasing non-equidistance leads to a merging of these into a single maximum near ~90° tuning width.

However, this transition occurs only at very high eccentricities (≥ 70°), where the model may be less reliable, and for non-equidistance values substantially larger than those observed empirically. The lower panels of C and D (bottom) summarize these results across eccentricities. The minimum sensitivity curves derived from empirical preferred directions fall within the range predicted by the model. Furthermore, the empirical average tuning widths lie close to the maxima of minimum sensitivity (averaged across eccentricities), while remaining suboptimal with respect to average sensitivity.

Taken together these results reinforce the reviewer's intuition that non-equidistance might lead to sensitivity curves with only one maximum. This intuition aligns with some of the analyses we performed while working on this project. Our idea was that the maximum of the minimal SSI at very small width results from a perfect distribution of the tuning curves in the ideal system, which is not robust to perturbations like non-equidistance of preferred direction. However, despite our efforts, we were unable to find clear and statistically robust evidence for this effect. We believe this likely reflects limitations in the available dataset, which are themselves a consequence of the experimental challenges associated with collecting these data.

That said, we fully agree with the reviewer regarding the importance of optic flow in visual encoding. In this context, we would also like to refer the reviewer to our response to Reviewer 2's comment concerning non-uniform priors, where we discuss related considerations and make use of the findings of Sabbah et al. in the construction of our models. We have included an analysis of the impact of eccentricity - and thus of non-uniform priors - in the discussion and supplementary section C.

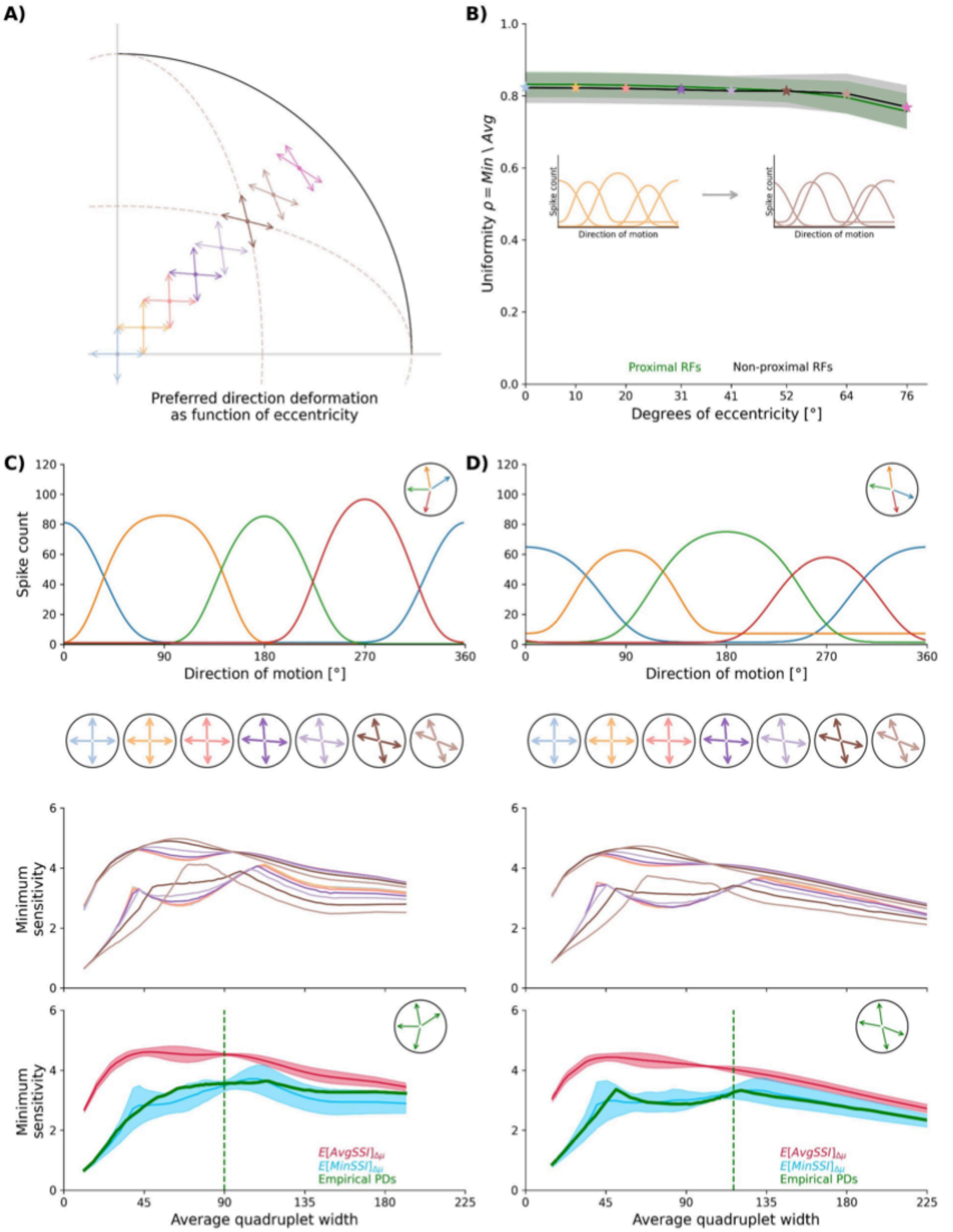

Figure 3: Uniformity and minimum sensitivity are impacted by eccentricity to different degrees.

A) Model of preferred direction deformation as function of eccentricity; **B)** Sensitivity uniformity as a function of eccentricity, for quadruplets of cells with proximal (green) or non-proximal (black) receptive fields; **C, D)** Minimum sensitivity as a function of eccentricity for 2 exemplary quadruplets. Top panels show the quadruplets' tuning curves. Arrows in insets show the color-coded preferred directions. Similarly, the row of arrows shows the preferred directions at 7 increasing degrees of eccentricity. Middle panels show minimum and average sensitivity changes associated to the modifications of preferred directions. Bottom panels show the averages of the minimum and average sensitivity over degrees of eccentricity, in red and blue, respectively. The green solid line shows the minimum sensitivity of the quadruplet with the original preferred directions. The green dashed line shows the quadruplet's average empirical tuning curve width.

3. Interaction between geometry and sensitivity:

How do receptive field overlap (point 1) and spacing irregularities of preferred directions (point 2) affect the relationship between minimum and average sensitivity? Quantifying this could provide mechanistic insight into how structural properties of the system shape tuning curve optimization.

This question reiterates our intuition about the potential mechanisms linking spatial organization and tuning properties.

As noted in our response to the first comment, Fig. A shows no significant difference in the relationship between minimum and average sensitivity (quantified via the uniformity index) when comparing quadruplets of cells with overlapping versus non-overlapping receptive fields. In addition, Fig. B indicates that receptive field overlap does not significantly influence the variation of the uniformity index across eccentricity.

We agree that the hypothesis that cells with overlapping receptive fields may be finely tuned to optimize encoding efficiency is both compelling and biologically plausible. However, this possibility is neither supported nor ruled out by the present analysis, and addressing it directly would require targeted experimental or modeling approaches beyond the scope of the current study.

Minor comments

- Figure 1: The current plotting order is confusing. Reordering panels from left to right and top to bottom would improve readability. In Fig. 1F, there is no cyan curve; this should be corrected.

Thanks, we have made the suggested modifications.

- Line 105: The phrase “in other words” should be removed, as the following statement does not follow directly from the preceding result.

Thanks, we have made the suggested modifications.

- Including predictions for retinas with three preferred directions would broaden the scope and applicability of the findings.

Apart from On-Off Direction Selective Retinal Ganglion cells, another type of direction-selective cell whose presence has been confirmed in the rabbit retina is the On-type direction selective cell. Unlike On-Off cells, which respond to increases and decreases in luminosity, On cells respond only to luminosity increases. They feature three preferred directions. (Oyster and Barlow, 1967 and Sivyer et al., 2010)

As ON DS cells recordings are difficult to obtain, we estimated the parameters of their tuning curves from Sivyer et al., 2010. Estimated Von Mises concentration for On cells was approximately 1.15 times larger than for On-Off cells, and the average firing rate approximately 66% of an equivalent On-Off cell (Sivyer et al., 2010, Fig.2). Each simulated experiment assigned cells to three groups to mimic three preferred directions and generated all unique triplets with one cell per group.

Tuning curve widening simulation results are shown in Fig. 4. On-cell tuning curves are less energetic and wider than On-Off cells (Fig. 4A, upper panel), producing wider individual sensitivities with fewer prominent peaks (middle panel). Compared to quadruplets, triplets show similar sensitivity minimum and average profiles as a function of average tuning curve width (Fig. 4A, bottom).

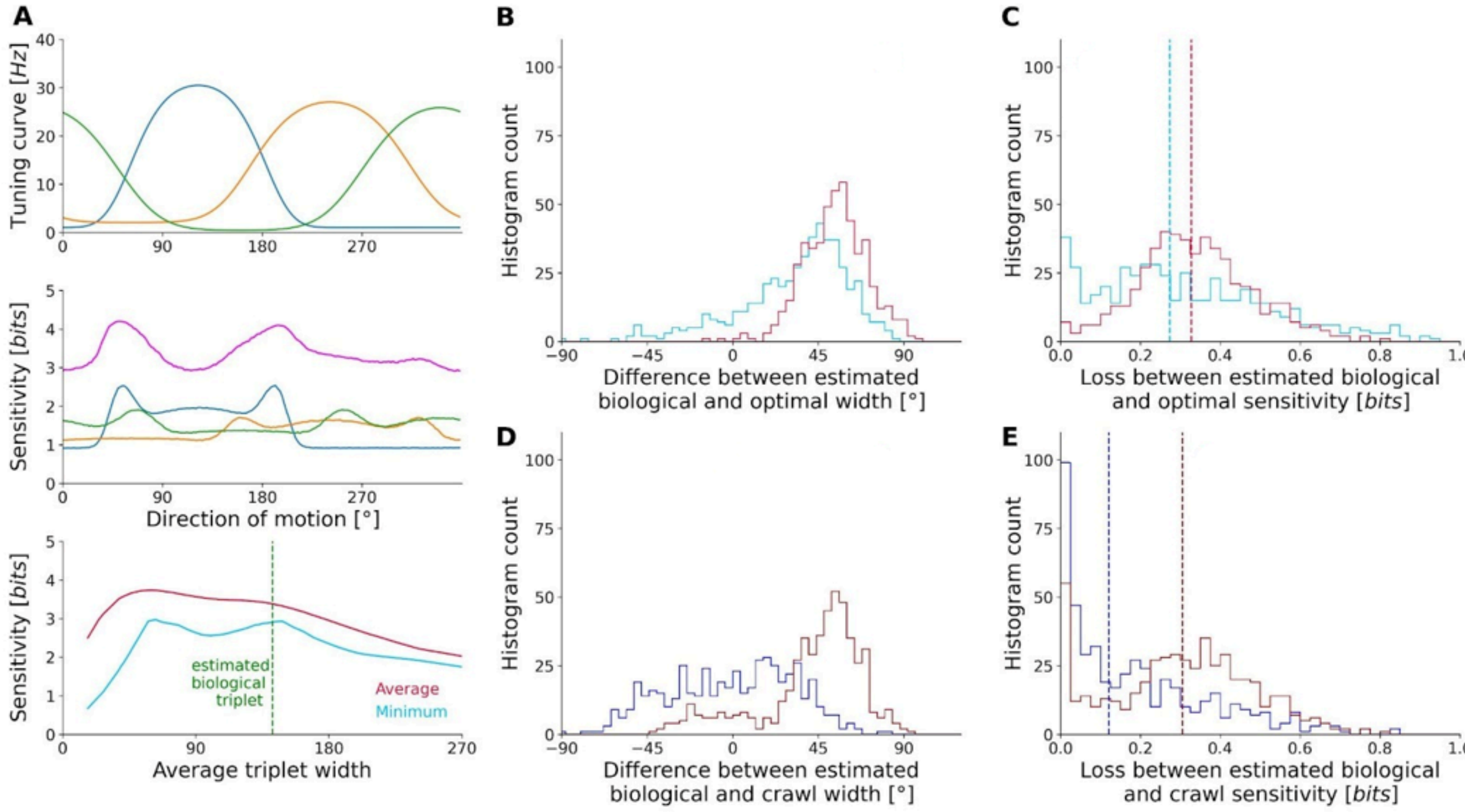

Figure 4: The sensitivity of a system with 3 preferred directions follows the predictions made by the MinMax sensitivity principle, especially when paired with the crawling algorithm.
A) Upper – example triplet of tuning curves; middle – individual and population sensitivities associated with the example triplet; bottom – behavior of the triplet sensitivity average and minimum (light red and light blue) as a function of simulated tuning curve widening. **B)** Difference between the estimated average "biological" triplet width and the width associated with the global maximum of the average and minimum sensitivity (light red and light blue), analogous to Fig.3C of the main manuscript. Most maxima occur at tuning curve widths approximately 45° narrower than the estimated biological width. Colored markers identify the bins containing the example triplet from panel A. **C)** Histograms of performance loss of estimated biological minimum and average sensitivity compared with associated maxima (light blue and light red), analogous to Fig.3D of the main manuscript. The trend is similar, but with a smaller peak around zero for the minimum sensitivity. **D)** Difference between the estimated average "biological" triplet width and the width associated with the crawl-obtained maximum of average and minimum sensitivity (dark red and dark blue), analogous to panel B. The histogram for the minimum is wider than its quadruplet counterpart. **E)** Histograms of performance loss of estimated biological minimum and average sensitivity compared with crawl-obtained maxima, analogous to panel C. Crawl-obtained widths favor maximizing the sensitivity minimum rather than the average, though the effect is less marked for triplets.

Global maxima analysis indicates that optimal widths for both criteria are ~45° narrower than the estimated biological widths (Fig. 4B). Loss histograms retain quadruplet trends, but neither minimum nor average can reliably be considered a relevant optimization criterion for these cells (Fig. 4C). Crawl procedure results show that optimal triplets' average widths are widely distributed around estimated biological widths, with lower performance loss for sensitivity minima versus averages. Initial parameter estimates were rough but trends and statistical behaviors persisted.

In conclusion, our analyses suggest that triplets of direction selective cells exhibit relatively uniform sensitivity and that the estimated biological tuning widths (which are believed to be broader for On DS RGCs than for On-Off DS RGCs) are more consistent with optimization of the minimum sensitivity than of the average sensitivity. Similarly, the crawling algorithm tends to converge toward widths associated with high minimum sensitivity, as observed for quadruplets. However, because these analyses rely on estimated tuning widths rather than direct physiological recordings, the conclusions should be regarded as preliminary. While the results are consistent with the trends observed in quadruplets and point in the same direction as our proposed framework, additional experimental data will be required to determine how strongly these principles apply to On DS RGC populations.
We have therefore included a brief mention of our results in the Discussion and this figure in the Supplementary Information.

Overall, this is a strong and thought-provoking manuscript that addresses an important question with clarity and rigor. Addressing the points above would further enhance the interpretability, generality, and impact of the work.

Thanks a lot! We really appreciate your positive comments on our manuscript.

Reviewer #2:

The manuscript from Paris et al. addresses an important question: are intermediate motion directions encoded less effectively than cardinals by direction-selective retinal ganglion cells? Especially since natural motion can occur at any angle. Using previously recorded rabbit ON–OFF DS RGCs, the authors fit tuning curves and use Stimulus Specific Information (SSI) to quantify direction-specific sensitivity in small populations ("quadruplets," one cell per subtype). They report that population sensitivity is nearly uniform across directions, and that empirical tuning widths sit near a local optimum that maximizes the minimum sensitivity (avoiding blind spots) rather than maximizing the average transmitted information (mutual information).

My major critiques to the manuscript are: Even though the study is motivated by natural stimuli, no natural stimuli are used, and the manuscript would benefit from a broader stimulus context (see major comment 1 & 2 below). The same dataset has been used in two other papers (Fiscella et al. 2015 & Franke et al. 2016); the novelty of the present work should therefore be clarified more clearly in the introduction (see major comment 5 below). Related to this point, the noise correlations found in Fiscella et al. 2015 and Franke et al. 2016 should be addressed (Comment 3). Additionally, it would be nice for the quadruplets to include the information of receptive field distance and overlapping receptive field (see comment 4). With these additions (or clear discussions of limitations), the manuscript's claims would be both clearer and more robust.

We thank the reviewer for their careful reading of the manuscript and their insightful comments.

Major comments

1) Natural vs. Artificial stimuli

The abstract and introduction emphasize natural stimuli and "everyday vision," but all analyses use artificial moving bars at a single speed/contrast. Please either (i) analyse a second, more naturalistic stimulus set, or (ii) state clearly in the abstract and introduction that conclusions are drawn from artificial bar motion and discuss scope.

The reviewer is right. We made the following changes in abstract, introduction and discussion.

ABSTRACT: … However, natural stimuli can move in any direction, and it remains unclear whether intermediate directions are encoded less effectively, or whether this cardinal organization nevertheless supports uniform direction encoding. Here, we combined previous electrophysiological recordings of DS cells presented with a moving light bar stimulus with an information-theoretic measure, the Stimulus Specific Information, to estimate the directional sensitivity of small populations of recorded neurons. This analysis revealed that DS RGC populations are uniformly sensitive across all directions of motion.

INTRODUCTION: …To demonstrate this result, we applied an information-theoretic analysis to previous retinal recordings in rabbit \cite{Fiscella15,Franke16}.

We begin by introducing and characterizing the dataset~~, in which, for~~ of electrophysiological recordings of DS RGC responses to repeated presentations of a moving bar stimulus. For quadruplets of DS RGCs - each comprising one cell per type - we observe that sensitivity is approximately uniform across motion directions. Quadruplets of DS RGCs - each comprising one cell per type - we observe that sensitivity is approximately uniform across motion directions.

We also included the analysis of a different stimulus (Section E of the new manuscript). See response to Reviewer 2's question 2 for details.

Also, the SSI is computed with a uniform stimulus prior $p(\theta)$ (line 261): it would be good to assess whether the main finding of uniform population sensitivity and the conclusion that the population prioritizes maximizing the minimum sensitivity rather than the average transmitted information holds under non-uniform priors (e.g., bias temporal-nasal motion statistics vs. superior-inferior motion). At minimum, discuss the expected impact.

We thank the reviewer for this question, which prompted us to examine more explicitly the role of non-uniform priors shaped by retinal geometry. The connection to optic flow statistics characterized by Sabbah et al. (*Nature*, 2017) suggested by reviewer 1 also motivated us to extend the analysis in this direction.

To address this point, we considered a simplified geometric model inspired by Sabbah et al., in which preferred directions vary systematically with eccentricity due to optic flow constraints. In this framework, each direction is expressed as a linear combination of "base vectors" tangent to ellipses defined by the naso-temporal and dorso-ventral axes, with magnitudes determined by the orthogonal projection of a hemispherical surface onto the retinal plane. As eccentricity increases, this construction produces progressively larger

deviations from equidistant direction configurations, consistent with experimental observations.

We assumed that the prior distribution over motion directions is uniform at the geometric center of the retina (panel A, blue arrows), and becomes non-uniform with increasing eccentricity (panel A, pink arrows). The corresponding priors are shown in panel B. Notably, at an eccentricity of approximately 75°, the prior becomes strongly bimodal. To probe the interaction between prior and likelihood, we focused on two representative quadruplets corresponding to extreme signal-to-noise regimes: one with low average spike count maxima (panel C) and one with high maxima (panel D). This choice reflects the dependence of our sensitivity measure on signal-to-noise ratio, and allows us to disentangle the contribution of the prior from that of the likelihood.

The resulting sensitivities are shown in panels E and F. The blue curves correspond to the sensitivity computed under a uniform prior Π_U , $I_{SSI}(\Pi_U; \theta)$, while the pink curves correspond to the sensitivity under a non-uniform prior Π_{NU}, $I_{SSI}(\Pi_{NU}; \theta)$. The dominant difference between these curves is a constant offset, which can be attributed to the difference in prior entropies: $\epsilon = H[\Pi_U] - H[\Pi_{NU}]$. To account for this, we define an adjusted sensitivity, $I_{SSI}(\Pi_{NU}; \theta) = I_{SSI}(\Pi_{NU}; \theta) + \epsilon$ (shown in black), which largely restores alignment with the uniform-prior case.

We also examined how the minimum sensitivity landscape depends on the prior by repeating the analysis of Section C in the main manuscript text under both priors. Panels G and H in Fig. 1 show the results for the two representative quadruplets. In both cases, the qualitative conclusion remains unchanged: the tuning configurations lie close to those that maximize minimum sensitivity, with only a small deviation from the global optimum.

Interestingly, for the high-firing-rate quadruplet (panel D), the sensitivity (panel F) and minimum-sensitivity curves (panel H) are nearly identical, differing primarily by a systematic offset attributable to the different prior entropies, as reflected in the near-complete overlap of the black and blue curves. This indicates that, in this regime, the shape of the sensitivity profile is largely determined by the likelihood. For the low firing-rate quadruplet, a small deviation is apparent, although its magnitude remains modest and does not alter the overall conclusions.

Overall, this analysis supports the view that, in the system considered here, prior-induced effects are secondary to those arising from the likelihood, particularly in regimes of high signal-to-noise ratio. Supplementary section C contains the previous analysis, and we have modified the Discussion by adding the following passage:

…Second, retinal neurons *in-vivo* are exposed to a broad range of stimulus statistics, which in our framework are reflected in the prior distribution over stimuli. Throughout this work, we assumed a uniform prior, corresponding to equal probabilities for all directions of motion. An important extension would be to investigate the robustness of our findings under non-uniform stimulus priors (Sabbah et al. 2017, *Nature*). For quadruplets of On-Off DS RGCs, our results indicate that the likelihood distribution (i.e. the tuning curves) provides the dominant contribution to sensitivity, while the prior distribution affects sensitivity only through a constant additive shift determined by the prior entropy (Suppl. section C)....

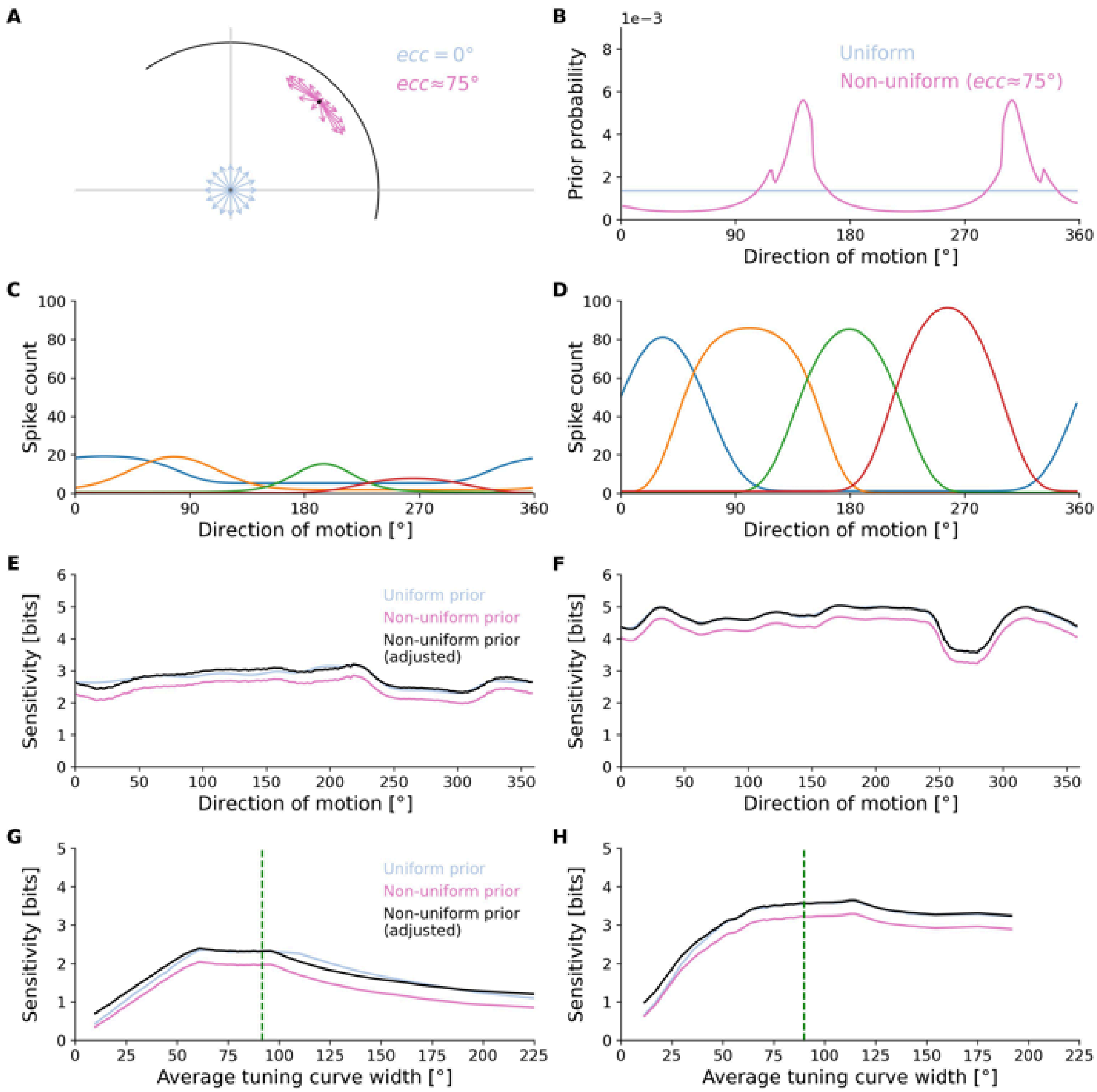

Figure 1: There is a modest impact of a non-uniform prior probability of direction of motion on quadruplet sensitivity, mostly due to a constant shift in prior entropy.

A) Simple geometric model of how the probability distribution of directions of motion changes with eccentricity. The arc represents a quadrant of the retina, the blue arrows represent the distribution of directions of motion in the retina's geometric center (equiprobability is represented by identical arrow length), while the pink arrows represent the modified non-uniform prior at an eccentricity of ~75°. **B)** The uniform and non-uniform priors obtained through the model in panel A, shown in blue and pink, respectively. **C,D)** The quadruplets with the lowest and highest average spike counts, respectively, from our population of quadruplets. **E,F)** The sensitivities of the weakest and strongest quadruplets, computed using the uniform prior (blue) and the non-uniform prior (pink). The adjusted sensitivity (black) is obtained by shifting the pink curve by the difference between the uniform and non-uniform prior entropies, to highlight the overlap with the uniform prior sensitivity and the contribution of the likelihood in its computation. **G,H)** The minimum sensitivities as a function of average tuning curve width (obtained as in Fig. 3 of the main text), computed using the uniform and non-uniform priors. The black lines are obtained using the non-uniform prior and then adjusted as in panels E and F.

2) Single stimulus set (only one speed, bar width etc.)

All results come from one stimulus set (fixed bar width, speed, contrast). Fiscella et al., the study from which the data originate, also uses different stimulus parameters (e.g. different speed and bar width). It would be nice to repeat the key analyses across bar widths/speeds and maybe also the “moving flashing square” stimulus used in Fiscella et al. This would directly test whether the idea of uniform population sensitivity and Max-Min sensitivity principle persists when tuning widths and gains shift with stimulus statistics.

We thank the reviewer for this suggestion, which motivated us to examine an additional dataset collected under different stimulus conditions to assess the generality of our conclusions. We thus tested the robustness of the observed sensitivity uniformity and the MaxMin principle across another dataset where the stimulus bar moves slower. Overall, the results from this additional dataset closely reproduce those reported in the main text and support the conclusions regarding sensitivity uniformity and the applicability of the MaxMin principle to On-Off DS RGC populations. We will report below the details of the analysis. On the same line, we invite the reviewer to appreciate the analysis we have done on On DS RGCs reported in the response to the last comment by reviewer 1 and now added to the Supplementary section.

To test the robustness of our results, we analyzed an additional dataset of HDMEA electrophysiological recordings from New Zealand albino rabbit On-Off DS RGCs, acquired across three independent experiments. In the original dataset presented in the main text, the stimulus consisted of a moving light bar with leading-edge widths of 0.5 mm and 1 mm, moving at 1.6 mm/s in 36 equally spaced directions. In the new dataset, the moving light bar had a leading-edge width of 0.5 mm, moved at 0.8 mm/s, and was presented in 16 equally spaced directions. Each direction was repeated 100 times per experiment.

Across the three experiments, 30 cells were recorded, of which 23 satisfied our inclusion criterion of a Flat-Topped Von Mises fit mean squared error below 15 (Methods). Because two of the three experiments lacked at least one preferred-direction class, complete quadruplets could not be formed within individual experiments. We therefore generated 270 quadruplets from the pooled population of 23 cells.

Panel A shows the tuning curves and associated directional sensitivity profile of an example quadruplet from the new dataset. Panel B shows the distribution of uniformity indices (minimum divided by average sensitivity) for the 270 quadruplets. Consistent with the original dataset, the uniformity index remained high, with a median value of approximately 0.76, and the distributions from the two datasets substantially overlapped. Panel C compares the performances of empirical quadruplets with globally optimal solutions in terms of minimum and average sensitivity. Losses relative to the global optimum were consistently smaller when evaluated using minimum sensitivity than average sensitivity. In the new dataset, the minimum sensitivity loss distribution was more strongly skewed toward zero than the average sensitivity distribution, indicating that empirical quadruplets more closely maximize minimum sensitivity than average sensitivity. Median losses in the new dataset were approximately 0.35 bits for minimum sensitivity and approximately 0.5 bits for average sensitivity. Panel D repeats the comparison using crawl-optimal rather than globally optimal solutions. As in the main text, crawl-optimal performances were substantially closer to the empirical observations than globally optimal performances, particularly when evaluated using minimum sensitivity. Negative values in panel D are due to noise at the local minimum sensitivity peaks. One noticeable negative value occurs in the old dataset (-0.24 bits), due to a local maximum of minimum sensitivity at very large average tuning curve widths. Panels E

and F compare empirical tuning widths with the widths maximizing sensitivity under globally optimal and crawl-optimal solutions, respectively. For both the original and new datasets, globally optimal average sensitivity was associated with narrow tuning curve widths, whereas

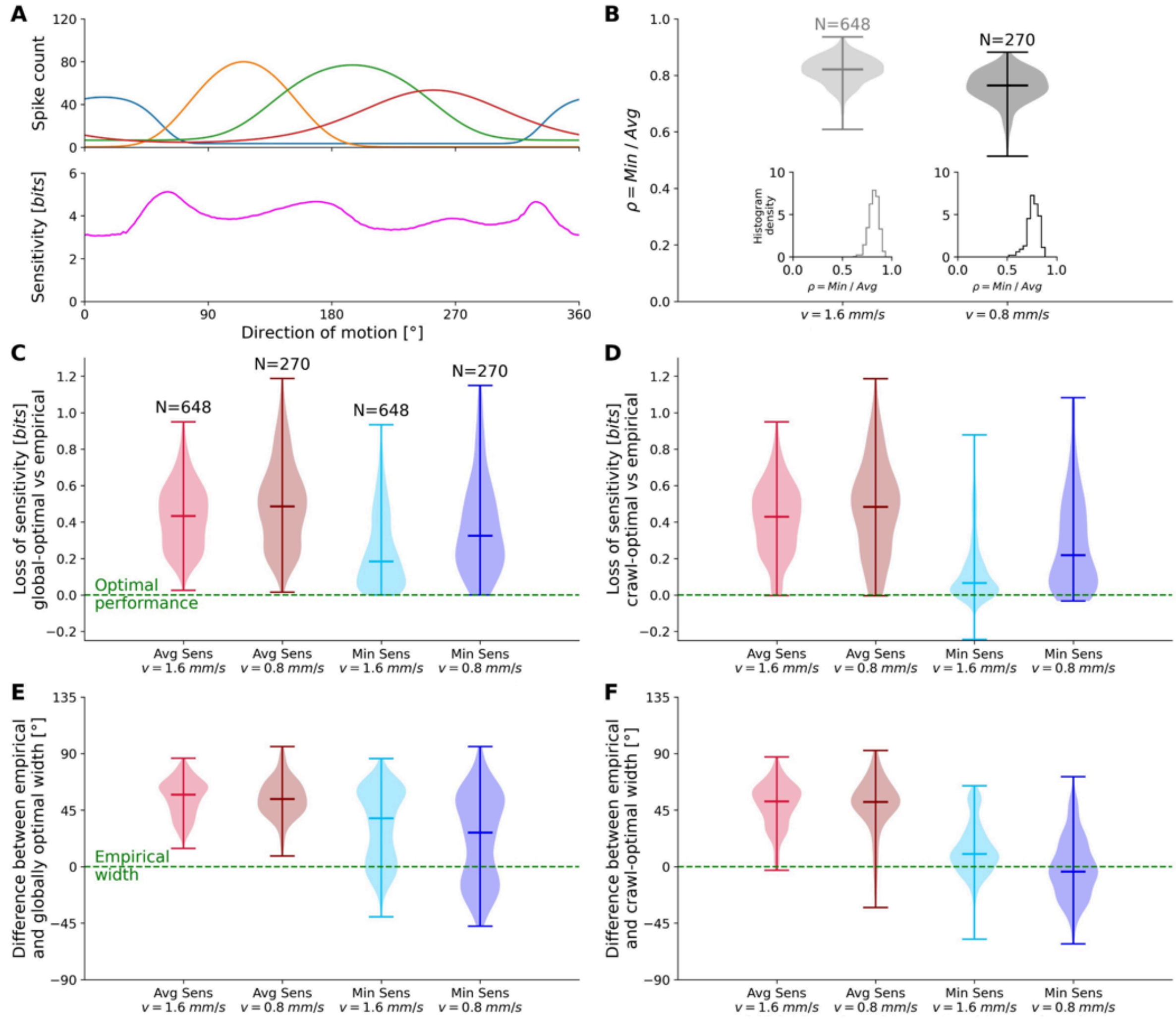

Figure 2: Comparison between the original and new datasets reveals robust sensitivity uniformity and preservation of the MaxMin principle under altered stimulus conditions
A) Example quadruplet from the new HDMEA dataset recorded using a light bar moving at 0.8 mm/s in 16 directions, together with its associated sensitivity profile. **B)** Uniformity index distributions from the original and new datasets substantially overlap. **C)** Violin plots showing sensitivity losses of empirical quadruplets relative to globally optimal solutions for minimum (blue) and average (red) sensitivity. Light colors correspond to the original dataset (1.6 mm/s), dark colors to the new dataset (0.8 mm/s). In both datasets, empirical quadruplets are closer to the optimum for minimum sensitivity than for average sensitivity. **D)** Sensitivity losses relative to crawl-optimal solutions, showing substantially smaller deviations from empirical performances than globally optimal solutions. **E)** Differences between empirical tuning widths and widths achieving globally optimal sensitivity. Average sensitivity is optimized at narrow widths, whereas minimum sensitivity also exhibits optima near empirical widths. **F)** Differences between empirical widths and crawl-optimal widths, showing convergence toward empirical widths when optimizing minimum sensitivity.

minimum sensitivity exhibited a bimodal structure, with optima occurring either at very narrow widths or near empirically observed widths. Importantly, the crawl algorithm combined with the MaxMin principle converged toward empirical tuning widths, with the crawl-optimal

minimum-sensitivity distributions concentrated near zero difference from the biological widths.

Together, these results demonstrate that sensitivity uniformity, the MaxMin principle as a coding strategy for On-Off DS RGC populations, and crawl-based recovery of empirically observed tuning widths remain robust across different stimulus statistics. We have added this result to the main text, as a new section E of the manuscript.

We also note that additional datasets acquired under other stimulus conditions are available. However, those recordings contain substantially fewer repetitions per condition, resulting in noisier tuning-curve estimates and less reliable sensitivity measurements. For this reason, we chose not to include those analyses in the present rebuttal.

3) Conditional independence assumption vs. noise correlations

The presented method assumes conditional independence within quadruplets (line 248-254), yet the same dataset has measured noise correlations that can affect coding/decoding, see e.g. Fiscella et al., 2015 who state that noise correlations are beneficial for coding of motion. And Franke et al., 2016 that show that noise correlation improves visual coding. Noise correlations are not discussed in the current manuscript. At minimum, discuss the previous findings and how this assumption might bias the conclusions. It would be nice to remove noise correlation and recompute population sensitivity and local maximum for minimum and average sensitivity curves (e.g. trial shuffling so that each cell's tuning curve in the quadruplets is calculated from different trials).

The reviewer is right, both in Fiscella et al 2015 and Franke et al 2016 noise correlations are shown to be helpful for coding/decoding, and it was a mistake by us to not discuss this point. Yet, even if we didn't mention it, we looked for their impact on our MaxMin principle in these data. The short answer is that the effect of noise correlation on our sensitivity is not strong enough to change its behavior for different directions, and thus to affect the results on uniform sensitivity or optimal width.

To showcase this effect, we focused on the forefront quadruplet of Franke et al. (*Neuron,* 2016) that was shown to showcase the effect of noise correlations:

1) We took the empirical distribution of the spike-counts of the four neurons for all the different directions.
2) We considered both the true distribution, and the one obtained after shuffling the trials to remove noise correlations.
3) We smoothed them in order to cope with the small number of repetitions, similar to what was done in Franke et al. 2016.
4) We estimated the SSI from the two distributions

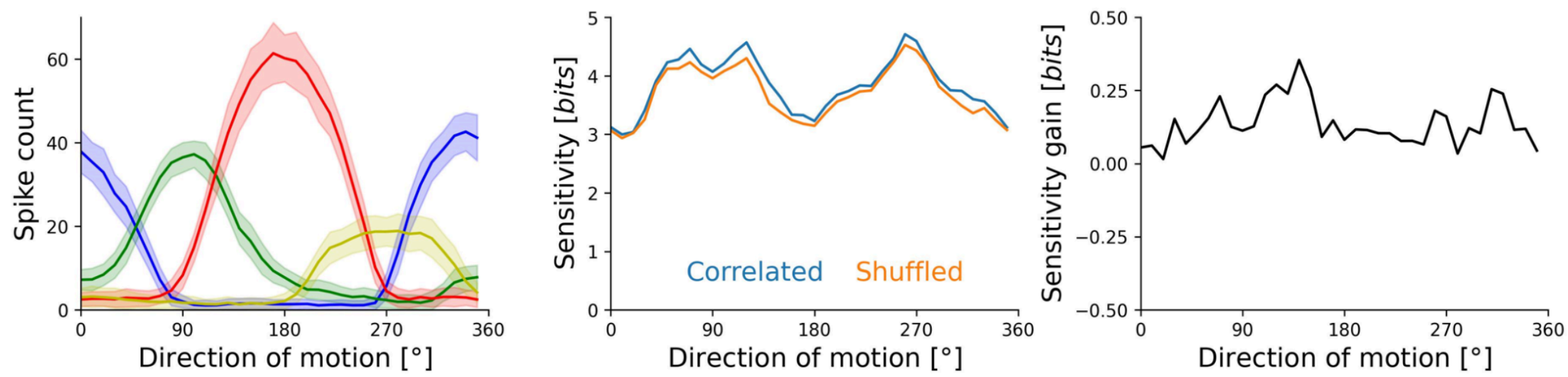

Figure 3: Impact of stimulus-dependent noise correlations on sensitivity in an example quadruplet.
A) Example quadruplet of tuning curves. Solid lines show average spike counts; shaded regions show the standard deviation of neuronal responses. **B)** Sensitivity estimates computed from correlated data and from shuffled (correlation-removed) data. **C)** Sensitivity gain attributable to stimulus-dependent noise correlations. The effects of noise correlations are modest but detectable.

As shown in Fig. 3 above, the sensitivity of the shuffled case is smaller, in accordance with Franke et al. 2016, but the difference, although not constant, is not big enough to change the uniformity across stimulus directions. Please note that our measure of sensitivity, the SSI, is logarithmically related to the one of Franke et al. 2016, which is based on Fisher information. This explains the different scale of our improvement compared to theirs. The same analysis carried with the Fisher information gives larger differences, but they don't affect uniformity as in our case. Noise correlations are a relevant feature of this system, which however does not impact the uniformity of sensitivity. We have added a comment about these results in the discussion.

4) Quadruplets and overlapping receptive fields

Quadruplets are motivated by tiling and minimal same-type overlap, implying that a small local stimulus is processed by one cell of each subtype. It seems that receptive-field (RF) locations are available from the dataset (from the flashing-square stimulus or electrode location from Fiscella et al.), it would therefore be informative to: -Tag quadruplets by RF overlap/distance and show whether the population sensitivity and Max-Min sensitivity principle differs for spatially co-localized vs. widely separated sets.

We thank the reviewer for this insightful question, which aligns with an analysis we performed in depth while preparing this manuscript. The short answer is that we don't see any significant difference between quadruplets made by proximal cells and non-proximal ones. See also the response to reviewer 1 question 1.

To address this point, we partitioned our population of quadruplets into "proximal" and "non-proximal" groups. Proximal quadruplets were defined as those composed of cells whose centers of mass were all within 300 μm of one another. Out of 648 randomly generated quadruplets, 84 satisfied this criterion. Panel A shows the distribution of uniformity indices (defined as the ratio between minimum and average sensitivity) for proximal (green) and non-proximal (black) quadruplets. The two distributions exhibit substantial overlap. A Kolmogorov–Smirnov test yields a p-value of 0.195 (> 0.05), indicating that we cannot reject the null hypothesis that the two samples are drawn from the same underlying distribution. Within the limits of our dataset, this suggests that receptive field overlap does not have a significant impact on sensitivity uniformity.

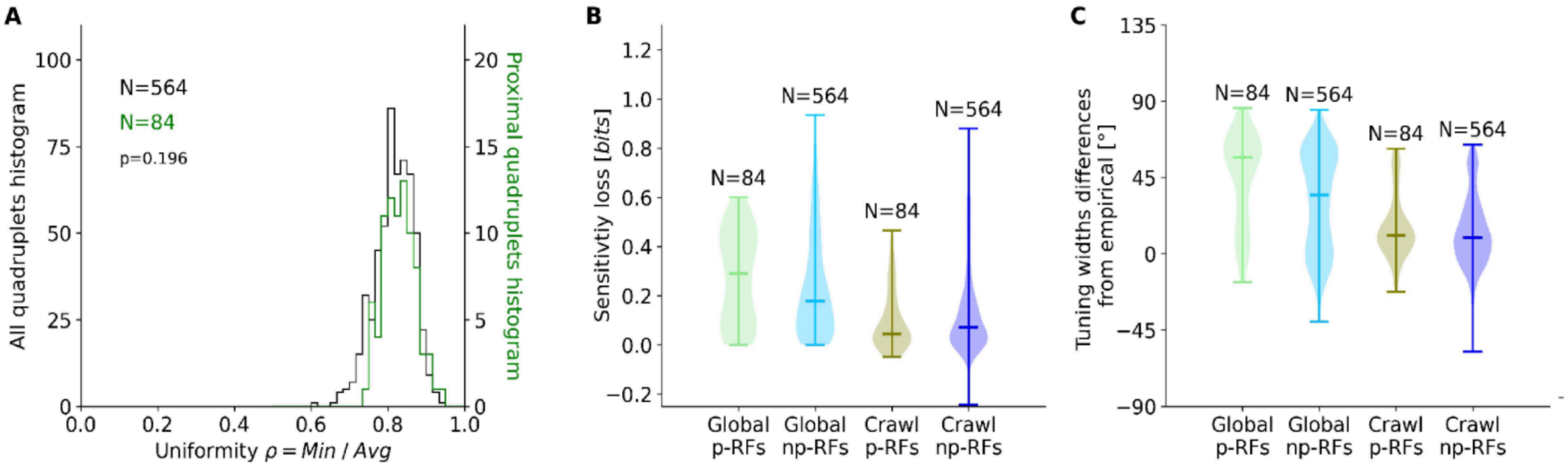

Figure 4: Receptive field overlap has a moderate impact on the uniformity of sensitivity and the minimum sensitivity as a function of average tuning curve width **A)** Uniformity distributions for quadruplet subpopulations with non-proximal (np-RFs, black) and proximal receptive fields (p-RFs, green). **B)** Violin plots of minimum sensitivity losses for empirical quadruplets relative to global (light) and crawl (dark) optima. Proximal quadruplets show larger losses at empirical average tuning widths compared to non-proximal quadruplets, whereas crawl optima converge near empirical performance regardless of receptive field position. **C)** Violin plots of width differences for global and crawl optima, separated by receptive field overlap. Global optima for proximal quadruplets favor narrow tuning widths, while non-proximal quadruplets display a more bimodal distribution; crawl optima remain close to empirical widths in both cases.

We further compared the dependence of minimum sensitivity on tuning curve width for proximal and non-proximal quadruplets. Panel B shows violin plots of the minimum sensitivity losses of empirical quadruplets relative to the global (light colors) and crawl (dark colors) optima. As expected, the globally optimized quadruplets achieve their highest minimum sensitivity either at very narrow tuning widths or at tuning widths that closely match those observed in the empirical quadruplets. In addition, the crawl algorithm consistently converges to solutions whose minimum sensitivity values are very similar to those of the empirical quadruplets, regardless of whether the receptive fields are proximal or non-proximal. This means that both proximal and non-proximal quadruplets indeed exhibit minimum sensitivity peaks at values similar to the performances achieved by the empirical quadruplets.

Panel C shows violin plots of width differences for the global and crawl optima of minimum sensitivity, separated by receptive field overlap. The global optima of proximal quadruplets are predominantly located at narrow average tuning curve widths, whereas non-proximal quadruplets display a more pronounced bimodal distribution. Nevertheless, the crawl algorithm converges to widths close to the empirical ones regardless of receptive field placement.

These results suggest that receptive field proximity has, at most, a limited influence on the minimum-sensitivity landscape. Both proximal and non-proximal quadruplets exhibited qualitatively similar behavior. Some differences between the distributions of optimal widths and performances were observed in the global optima, which may indicate a modest effect of receptive field position on sensitivity. However, the available data do not allow us to determine whether these differences reflect a genuine effect. The number of spatially proximal quadruplets was substantially smaller than in the full dataset (84 versus 564), limiting statistical power. In addition, individual cells were reused across multiple quadruplets, introducing correlations between samples and reducing the effective sample size. Consequently, while the data allow for a possible influence of receptive field proximity on

minimum sensitivity, they do not provide sufficient evidence to draw firm conclusions regarding its role.

We have added a mention of this analysis in the Discussion, and have included it in Supplementary section B.

-Related to the quadruplets. Cafaro et al., 2020, J.Neuroscience showed that decoding works well only when large populations of DS cells are used. Real visual scenes will activate many DSCs at once. It would be nice to use the same tuning-curve approach with a larger population (8,16,32 DS cells, preserving mosaics) and recompute SSI and local optima. Does the population sensitivity and Max-Min sensitivity principle change when using more than 4 cells?

We thank the reviewer for raising this point. To clarify the relationship between population size, uniformity of sensitivity and the MaxMin principle, we now include additional analyses for 8- and 16-cell populations, which we find consistent with the results previously shown for quadruplets.

We randomly generated 200 8-cell and 200 16-cell populations from experimentally measured tuning curves. Due to the limited number of available cells per preferred direction per experiment, some cells might be taken twice for construction 8-plets or 16-plets. In each case, populations were constructed with a fixed number of cells per preferred direction (2 and 4, respectively). Fig. 5A shows an example 8-cell population (top), together with its corresponding sensitivity profile (bottom). Fig. 5B shows the analogous construction for a 16-cell population. As population size increases, sensitivity minima become less pronounced, consistent with improved tiling of the stimulus space by the tuning curves. In addition, because more cells contribute to encoding each stimulus, the overall (average) sensitivity increases.

Fig. 5C shows that uniformity remains high for both 8- and 16-cell populations, with a reduced fraction of low-uniformity cases compared to 4-cell populations. The median uniformity indices for 4-, 8-, and 16-cell populations are quite compatible: 0.82, 0.82, and 0.83, respectively.

We next examined the distributions of performance losses relative to globally optimal minimum- and average-sensitivity solutions, as a function of average tuning curve width, and compared these to the performance of populations constructed from empirical tuning curves. Minimum- and average-sensitivity results are shown in blue and red, respectively. For reference, the first two violin plots in Fig. 5D reproduce the corresponding histograms from Fig. 3D of the main text. As in the case of quadruplets, 8- and 16-cell populations exhibit smaller losses relative to the globally optimal minimum sensitivity than relative to the globally optimal average sensitivity. Notably, as population size increases, the global optimum of average sensitivity increases, leading to a larger loss relative to empirical tuning curve widths

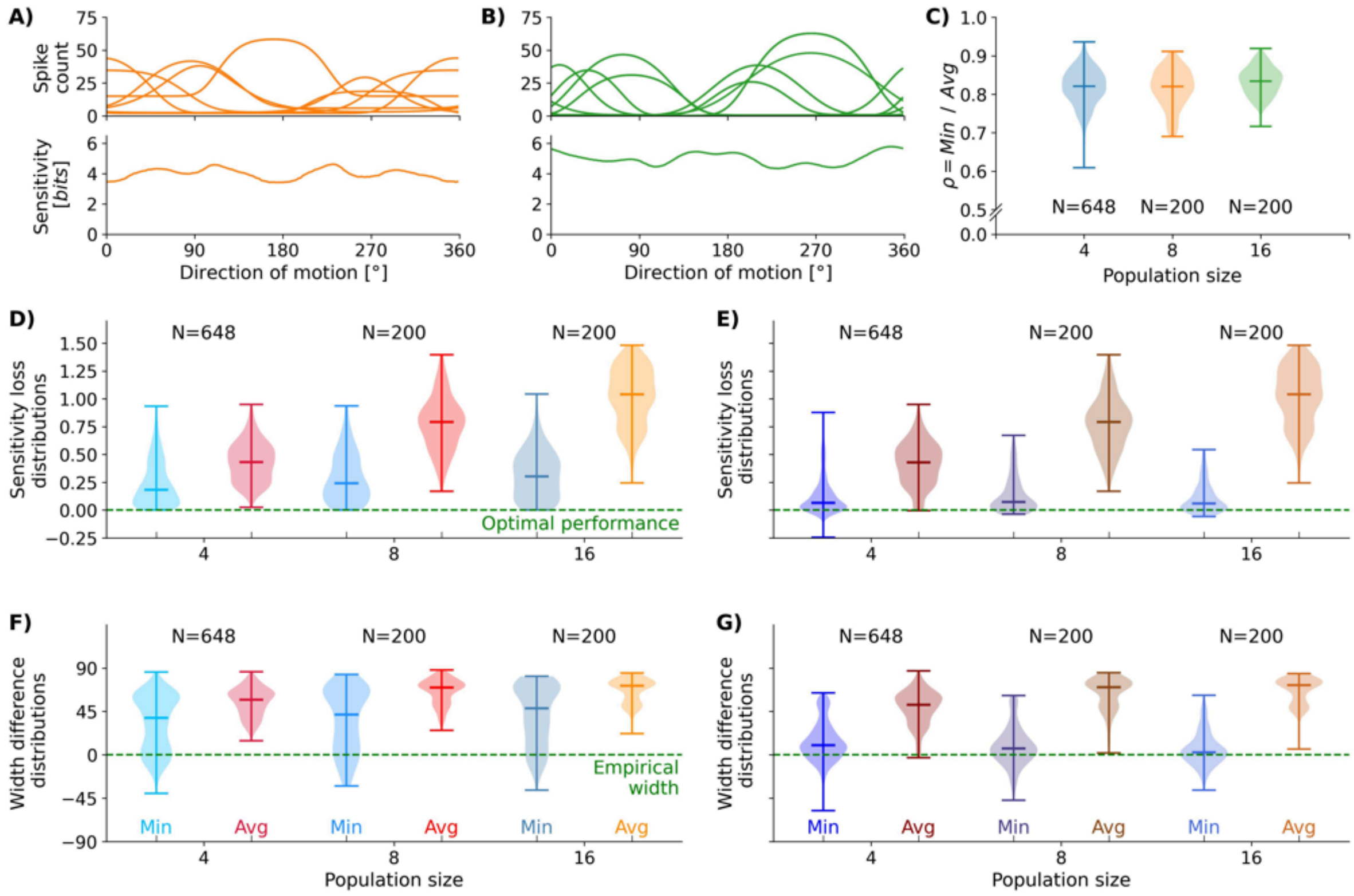

Figure 5: Repeated analyses for populations of 8 and 16 cells suggest the MaxMin principle and crawling algorithm converge to empirical minimum sensitivity performances and empirical tuning curve widths, like for quadruplets **A)** Example 8-cell population: two neurons per preferred direction (upper) and corresponding sensitivity profile (lower). **B)** As in panel A, but for a 16-cell population with four neurons per preferred direction. **C)** Uniformity indices for 4-, 8-, and 16-cell populations (blue, orange, green). Variability in uniformity decreases with increasing population size. **D)** Minimum (blue) and average (red) sensitivity losses relative to global optima. Empirical populations are closer to optimal under the minimum-sensitivity objective. **E)** The crawl algorithm converges toward empirical configurations along the minimum-sensitivity landscape, while identifying non-empirical global optima under the average-sensitivity objective. Although average-sensitivity optima deviate further from empirical configurations, the crawl still recovers empirical minimum-sensitivity solutions. **F)** Distributions of differences between empirical tuning widths and globally optimal widths for 8- and 16-cell populations mirror those of quadruplets. Narrow widths optimize average sensitivity, whereas minimum sensitivity is achieved across a broad range of widths, many close to empirical values. **G)** The crawl algorithm recovers empirically consistent tuning widths along the minimum-sensitivity landscape for 4-, 8-, and 16-cell populations.

under this objective. Nevertheless, across all population sizes, empirical configurations remain closer to the global optima under the minimum-sensitivity criterion (Fig. 5D).

This trend is further supported by the crawl algorithm (Fig. 5E), which preserves the magnitude of the loss relative to the optimal average-sensitivity solution, while converging toward configurations consistent with empirical minimum-sensitivity performance. Minor negative values in Fig. 5E arise from small numerical fluctuations near the minimum-sensitivity peak, with the exception of one quadruplet exhibiting a local maximum

at very large widths; this local maximum is approximately 0.25 *bits* lower than the performance at empirical tuning curve widths.

With respect to tuning curve widths, 8- and 16-cell populations show global optima for minimum sensitivity both at relatively narrow widths (approximately 45° narrower than empirical values) and at widths close to empirical measurements, as shown in the blue distributions in Fig. 5F. In contrast, global optima of average sensitivity consistently occur at narrow tuning widths across population sizes. However, when optimizing under the minimum-sensitivity objective, the crawl algorithm converges to empirically consistent tuning widths (Fig. 5G), while converging to distinct non-empirical optima under the average-sensitivity objective.

Together, these results further support the MaxMin principle as a candidate organizing rule for On-Off DS RGCs, and indicate that the crawl algorithm can be a viable principled route to recovering empirically observed tuning curve widths.

We have added this analysis in Supplementary section D, and added the following passage in the Discussion:

… Finally, a stimulus sweeping over the retina will most likely activate many DS cells at once. Therefore, a complete understanding of On-Off DS RGCs would require extending the present analysis beyond quadruplets of cells and investigating the sensitivity of larger neuronal populations. A similar behavior was observed in simulations of populations of 8 and 16 cells, which showed largely the same sensitivity trends as quadruplets, suggesting that our main conclusions may generalize to larger populations (Suppl. section D). …

5) Novelty relative to Fiscella (2015)

Fiscella et al. already showed that four broad DS types give good coverage and that adding types yields limited gains. Please clarify the novelty of your method compared to their work up front (Introduction).

The reviewer is right, we compared their results with ours only in the discussion, and not with the necessary depth.

From a functional perspective, our results complement and enrich their findings. They also find that the organization of preferred direction along cardinal axes leads to a quite uniform decoding error (their Fig. 3C), something in line with the uniformity of sensitivity we reported in Fig. 2, but they stop after a few examples, without discussing it as a general principle. They also performed an analysis by stretching the tuning curves similar to ours (compare Fig.3 and their Fig. 7), finding that the optimal corresponding to the minimum of the maximal error - instead of average error - is closer to the empirical tuning curves. This aligns with our maximum of minimal sensitivity results, which we however extend and complete via the crawling procedure, making sense of the observed discrepancy with optimal conditions, and which in our opinion has the deep biological implications that we explained in the discussion.

From a theoretical perspective, instead of working using decoding, we approached the problem from the encoding perspective using information theory. Although more abstract, this allowed us to link and contextualize our results within the efficient coding theory, which we generalize with our MaxMin neuronal principle.

Thanks to the referees suggestions, we now show that these results are robust across different stimuli and prior distribution, and probably generalize to ON DS cells with three preferred directions.

We added these arguments in both the Introduction and Discussion.

INTRODUCTION:
We show that the cells exhibit tuning curve widths associated with near-optimal coding performance, rather than the theoretical optimum predicted by our framework, suggesting that factors beyond the optimization criterion considered here may contribute to shaping their tuning properties. Our results stem from the findings of Fiscella et al. (2015) by recasting the problem within an information-theoretic framework, linking the observed tuning widths to a MaxMin coding principle that generalizes Efficient coding and explains the near-uniform sensitivity across motion directions. Finally, our crawling procedure explains the discrepancy between empirical and theoretically optimal tuning widths, revealing a near-optimal regime.

DISCUSSION:
While perfectly spaced and more numerous preferred directions lead to lower decoding error, Fiscella et al. (2015) propose that the upgrade might not be conducive to a net improvement, considering the resource cost necessary to achieve it. From a functional perspective, our results complement those of Fiscella et al. (2015): their analyses also point to uniform decoding performance and favor tuning widths that minimize the worst-case error, consistent with our Max-Min principle. By adopting an information-theoretic encoding framework, we further connect these findings to efficient coding theory and show that they are robust across stimuli and prior distributions. Our results regarding uniform sensitivity for a system with cells selective to a discrete number of preferred stimuli align with Theunissen et al. (1991), even though they worked on a completely different biological system (the cricket cercal system).

Minor comments

1. Tone/phrasing (Introduction and discussion): statements about "good enough coding" rather than "optimal" and "physiologically too costly" (line 70-71) could be misread. It's hard to tell what is too costly, especially since there are no cost analyses presented here—consider softening.

We thank the reviewer for this comment. We have modified the introduction as follows:
…In other words, the widths and preferred directions of the population are arranged such that the system avoids having any motion directions that are encoded significantly worse than others, thereby preventing the emergence of a kind of "blind spots".
~~Finally, we show that the cells converge on parameters that yield a "good enough" coding performance, rather than an optimal one, which may be physiologically too costly to achieve~~. Finally, we show that the cells exhibit tuning curve widths associated with near-optimal coding performance, rather than the theoretical optimum predicted by our framework, suggesting that factors beyond the optimization criterion considered here may contribute to shaping their tuning properties.

2. Abstract clarity (line 19-20): consider simpler language connecting "maximizing minimum sensitivity" to "avoiding blind spots." This helps non-specialists.

We thank the reviewer for his comment. We have modified the abstract as follows:
…Simulations with artificially modified tuning curve widths revealed that DS cells prioritize avoiding pronounced drops in sensitivity over maximizing the average transmitted

information. ~~Maximizing this minimal sensitivity may therefore be a principle to understand the organization of sensory systems.~~ This property reduces large deficits in sensitivity for certain directions, thereby limiting potential “blind spots” in the encoding of motion. More generally, our results raise the possibility that maintaining a high minimum sensitivity could be one factor influencing the organization of sensory representations.

3. Intro citations: where macaque retina is mentioned (line 37), ensure the macaque RGC citation appears (it should be [2,6-8]). Generally, check all citations carefully.

We thank the reviewer for his careful reading. We have made the necessary modifications.

4. Figure 1: Results text and legend show N = 82 cells for distributions, whereas Methods report n = 90 included. Please explain in the method why 8 cells were additionally excluded for the analysis.

Out of the initial 90 cells, 6 cells were excluded because the Flat-Topped von Mises fit had a Mean Square Error larger than 15, and an additional 2 were excluded because they had Full Width at Half-Maximum larger than 180. The suggested modification has been done to the Methods section.

5. Fig 1C: explain the bimodal distribution of the maximum firing rate. Does it come from different experiments or different subtypes?

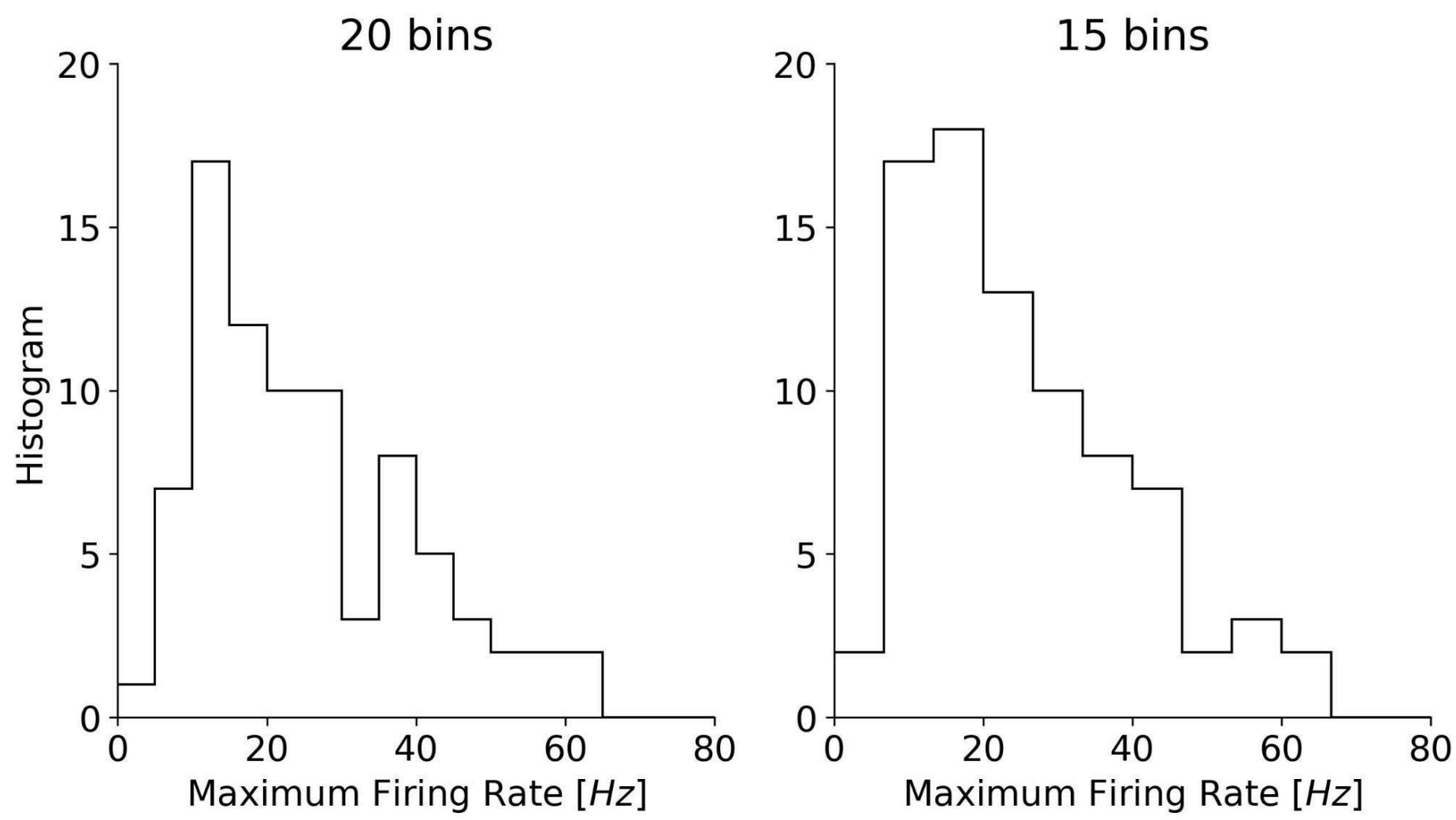

The bimodal distribution in Fig.1C comes from setting the number of histogram bins to a too high number, namely 20 bins (left panel). When setting the binning to a more reasonable number for a dataset of 82 cells, namely 15 bins, the peak at high firing rates disappears (right panel). We have substituted the right panel in Fig.1C.

6. Fig. 1E: The rVM vs. FTVM comparison—ensure colors/line styles match legend. It seems to me that the solid black line (rVM) fits the tuning curves much better than the dashed line (FTVM), especially for example 2. This appears to contradict the text claiming FTVM fits better—please check for legend/style mismatches or plotting errors.

Indeed, there was an error in the text: the rVM fit was supposed to be dashed, while the FTVM was supposed to be solid.

This error has been amended.

7. Fig 1F: The color cyan for rVM is missing in the plot, however it's supposed to be cyan as written in the legend. Please add the cyan color in the plot.

Similarly to the previous point, there was an error in the text: the lines relative to the rVM fit were supposed to be dashed, black lines, not cyan.

This error has been amended.

8.Fig. 3A: Middle/right panels are hard to see (both top for tuning curve and lower for sensitivity). Please adapt the y axis limits or make larger insets/zoom. In the current version one cannot see anything. Axis labels for tuning curve and sensitivity (Hz and bits) appear to be missing—please add.

The reviewer is right, that schematic was not very readable. We improved it in the new manuscript by zooming into the y axis of the upper panels in order to make the tuning curves more visible. The y axis for the bottom panels containing sensitivity plots were kept the same in order to show how the sensitivity decreases and flattens as tuning curve width increases.

9. Fig 3C: Explain more clearly the bimodal distribution in Fig. 3C (minimum sensitivity, blue).

The following modification has been applied to the text in Section C: "Widths of On-Off DS RGC tuning curves are tuned to maximize sensitivity minima".

…The minimum sensitivity can present either 1 or 2 clearly visible local maxima (exemplified in Fig.3B, blue lines)~~, located close to either the empirical or relatively much narrower widths~~. These maxima occur at tuning-curve widths that are either substantially narrower than the empirical values (left peak in the upper panel of Fig. 3B) or close to the empirical widths (right peak in the upper panel of Fig. 3B). Consistently, when studying all the N=648 possible quadruplets, the histogram of the widths corresponding to the optimal minimum sensitivity is bimodal, ~~with two peaks centered at values similar to or smaller than the empirical widths (blue histogram in Fig3C)~~ with peaks occurring at widths similar to the empirical ones (left peak of blue histogram in Fig.3C) and widths occurring at much narrower widths (right peak of blue histogram in Fig.3C).
Although the globally optimal average tuning curve width for a given quadruplet may be much narrower than the empirical one, the resulting difference in minimum sensitivity is typically small….

…These results suggest that empirical quadruplets are tuned to maximize minimum sensitivity rather than Mutual Information: a substantial number of quadruplets have an empirical width that achieves a performance close to the local maximum along the minimum sensitivity, while at the same time leading to a large loss in terms of average sensitivity (Mutual Information). Nevertheless, ~~the~~ many quadruplets seem to reach a near-optimal minimum sensitivity, rather than achieving the globally optimal configuration….
