# Supplementary material for: Direction-selective retinal ganglion cells encode motion direction uniformly, despite having discretely distributed cardinal preferences
Source: PLoS Comput Biol. 2026 Nov 2;22(11):e1014872. doi: 10.1371/journal.pcbi.1014872 (PMC13739222; doi:10.1371/journal.pcbi.1014872)
Supplement: Peer review history

# Author comment: Author response to Decision Letter 0

**DOI:** 10.1371/journal.pcbi.1014872.r001

21 Jun 2026

Attachment Submitted filename: Review_DS_Paris.pdf

# Peer review: Decision Letter 0

**DOI:** 10.1371/journal.pcbi.1014872.r002

**Authors:** Marieke van Vugt, William Redman

22 Sep 2026

Dear Mr Paris,

We are pleased to inform you that your manuscript 'Direction-selective retinal ganglion cells encode motion direction uniformly, despite having discretely distributed cardinal preferences' has been provisionally accepted for publication in PLOS Computational Biology.

Before your manuscript can be formally accepted you will need to complete some formatting changes, which you will receive in a follow up email. A member of our team will be in touch with a set of requests.

Please note that your manuscript will not be scheduled for publication until you have made the required changes, so a swift response is appreciated.

IMPORTANT: The editorial review process is now complete. PLOS will only permit corrections to spelling, formatting or significant scientific errors from this point onwards. Requests for major changes, or any which affect the scientific understanding of your work, will cause delays to the publication date of your manuscript.

Should you, your institution's press office or the journal office choose to press release your paper, you will automatically be opted out of early publication. We ask that you notify us now if you or your institution is planning to press release the article. All press must be co-ordinated with PLOS.

Thank you again for supporting Open Access publishing; we are looking forward to publishing your work in PLOS Computational Biology.

Best regards,

William Redman

Academic Editor

PLOS Computational Biology

Marieke van Vugt

Section Editor

PLOS Computational Biology

***********************************************************

All 3 reviewers were very enthusiastic on the quality of the manuscript after the revision and all 3 recommend accept. Please note the minor suggestions made by Reviewer 3 and consider making adjustments to address them, where appropriate.

Reviewer's Responses to Questions

**Comments to the Authors:**

**Please note here if the review is uploaded as an attachment.**

Reviewer #1: The authors have clearly put substantial effort into addressing all of the points raised. They have responded carefully and comprehensively to each comment, including additional analyses, figures, and revisions to the text that have significantly strengthened the manuscript. I am satisfied that all of my concerns have been fully addressed and I have no further comments. The manuscript has improved substantially and is now very clear and compelling.

Reviewer #2: Thank you very much for your thorough and thoughtful response to my comments. I appreciate the care with which you addressed the points raised, especially through the additional analyses and modeling of eccentricity effects and receptive field overlap.

I found your responses clear and convincing, and I have no further comments.

Reviewer #3: This paper seeks to address how the retina’s code for direction of motion can fill in the gaps between the non-uniform tuning peaks of retinal ganglion cells, enriched at four cardinal directions of motion, anchored on the animal’s egocentric axes. The analyses explore other kinds of readout objectives and show how a principal of minimum sensitivity (i.e. no gaps in motion direction encoding) explains the data well. This suggests, compellingly, that the eye has been organized to encode motion directions uniformly despite the asymmetries in tuning curve peak distributions.

Stepping into the review of this paper after the first rounds of revisions, I’m primarily struck by the thoroughness and care the authors have taken to address the concerns of the previous reviewers. The re-analysis, new simulations, and particularly the new dataset recorded combine to strengthen and extend the paper’s results and impact. I have no new major comments and believe this paper will appeal to the neuroscience community, broadly.

I have a few minor comments on some of the things a reader coming to this with fresh eyes might miss:

Include a sentence somewhere connecting intuitions the reader might have about Fisher information (another local measure of stimulus sensitivity) peaks on the flanks of tuning curves and the structure seen in the sensitivity curves. This is place where a general reader, unfamiliar with SSI, will need a little more guidance on how to understand and frame the current results.

If journal formatting constraints allow, add a short sentence at the beginning of each figure caption that summarizes the main takeaway of the figure.

Figure S5: label the magenta curve in the middle panel of A, and label the curves in B/C and D/E in the whitespace at the top of those panels.

**********

**Have the authors made all data and (if applicable) computational code underlying the findings in their manuscript fully available?**

The PLOS Data policy requires authors to make all data and code underlying the findings described in their manuscript fully available without restriction, with rare exception (please refer to the Data Availability Statement in the manuscript PDF file). The data and code should be provided as part of the manuscript or its supporting information, or deposited to a public repository. For example, in addition to summary statistics, the data points behind means, medians and variance measures should be available. If there are restrictions on publicly sharing data or code —e.g. participant privacy or use of data from a third party—those must be specified.

Reviewer #1: **No:**  Currently the code and data is not available, there is no link. The authors wrote: Code used for this publication will be made available on GitHub

Reviewer #2: Yes

Reviewer #3: Yes

**********

PLOS authors have the option to publish the peer review history of their article (what does this mean?). If published, this will include your full peer review and any attached files.

If you choose “no”, your identity will remain anonymous but your review may still be made public.

**Do you want your identity to be public for this peer review?**  For information about this choice, including consent withdrawal, please see our Privacy Policy.

Reviewer #1: No

Reviewer #2: No

Reviewer #3: No

# Editor report: Acceptance letter

**DOI:** 10.1371/journal.pcbi.1014872.r003

**Authors:** Marieke van Vugt, William Redman

PCOMPBIOL-D-26-01511

Direction-selective retinal ganglion cells encode motion direction uniformly, despite having discretely distributed cardinal preferences

Dear Dr Paris,

I am pleased to inform you that your manuscript has been formally accepted for publication in PLOS Computational Biology. Your manuscript is now with our production department and you will be notified of the publication date in due course.

The corresponding author will soon be receiving a typeset proof for review, to ensure errors have not been introduced during production. Please review the PDF proof of your manuscript carefully, as this is the last chance to correct any errors. Please note that major changes, or those which affect the scientific understanding of the work, will likely cause delays to the publication date of your manuscript.

Soon after your final files are uploaded, unless you have opted out, the early version of your manuscript will be published online. The date of the early version will be your article's publication date. The final article will be published to the same URL, and all versions of the paper will be accessible to readers.

For Research, Software, and Methods articles, you will receive an invoice from PLOS for your publication fee after your manuscript has reached the completed accept phase. If you receive an email requesting payment before acceptance or for any other service, this may be a phishing scheme. Learn how to identify phishing emails and protect your accounts at https://explore.plos.org/phishing.

Thank you again for supporting PLOS Computational Biology and open-access publishing. We are looking forward to publishing your work!

With kind regards,

Sharmila Kamatchi

PLOS Computational Biology | Carlyle House, Carlyle Road, Cambridge CB4 3DN | United Kingdom ploscompbiol@plos.org | Phone +44 (0) 1223-442824 | ploscompbiol.org | @PLOSCompBiol
